# Targeting antioxidant pathways with ferrocenylated N-heterocyclic carbene supported gold(i) complexes in A549 lung cancer cells†
†Electronic supplementary information (ESI) available. CCDC 1419940 and 1419941. For ESI and crystallographic data in CIF or other electronic format see DOI: 10.1039/c5sc03519h


**DOI:** 10.1039/c5sc03519h

**Published:** 2015-10-29

**Authors:** J. F. Arambula, R. McCall, K. J. Sidoran, D. Magda, N. A. Mitchell, C. W. Bielawski, V. M. Lynch, J. L. Sessler, K. Arumugam

**Affiliations:** a Department of Chemistry , Georgia Southern University , Statesboro , Georgia 30460 , USA . Email: jarambula@georgiasouthern.edu ; Tel: +1-912-478-2346; b Department of Chemistry , St. Bonaventure University , 3261 West State Road , New York , 14778 , USA; c Lumiphore, Inc. , Berkeley , California 94710 , USA; d Department of Health Sciences , Gettysburg College , Gettysburg , PA 17325-1400 , USA; e Center for Multidimensional Carbon Materials (CMCM) , Institute for Basic Science (IBS) , Ulsan 689-798 , Republic of Korea; f Department of Chemistry and Department of Energy Engineering , Ulsan National Institute of Science and Technology (UNIST) , Ulsan 689-798 , Republic of Korea; g Department of Chemistry , University of Texas at Austin , Austin , Texas 78712 , USA; h Department of Chemistry , Wright State University , 3640 Colonel Glenn Hwy , Dayton , Ohio 45435 , USA . Email: kuppuswamy.arumugam@wright.edu

## Abstract

Ferrocene containing N-heterocyclic carbene (NHC) ligated gold(i) complexes of the type [Au(NHC)_2_]^+^ were prepared and found to be capable of regulating the formation of reactive oxygen species (ROS) *via* multiple mechanisms. Single crystal X-ray analysis of bis(1-(ferrocenylmethyl)-3-mesitylimidazol-2-ylidene)-gold(i) chloride (**5**) and bis(1,3-di(ferrocenylmethyl)imidazol-2-ylidene)-gold(i) chloride (**6**) revealed a quasi-linear geometry around the gold(i) centers (*i.e.*, the C–Au–C bond angle were measured to be ∼177° and all the Au–C_carbene_ bonds distances were in the range of 2.00 (7)–2.03 (1) Å). A series of cell studies indicated that cell proliferation inhibition and ROS generation were directly proportional to the amount of ferrocene contained within the [Au(NHC)_2_]^+^ complexes (IC_50_ of **6** < **5** < bis(1-benzyl-3-mesitylimidazol-2-ylidene)-gold(i) chloride (**4**)). Complexes **4–6** were also confirmed to inhibit thioredoxin reductase as inferred from lipoate reduction assays and increased chelatable intracellular zinc concentrations. RNA microarray gene expression assays revealed that **6** induces endoplasmic reticulum stress response pathways as a result of ROS increase.

## Abstract 2

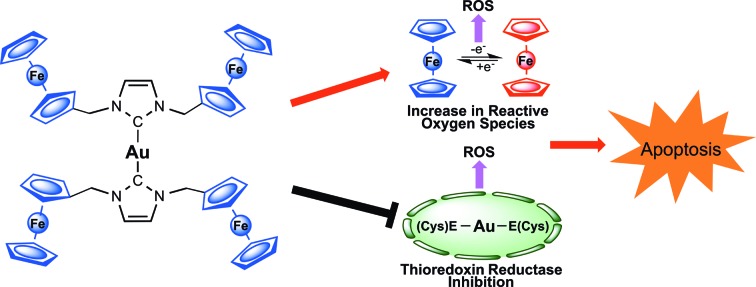
Ferrocenylated-Au(i) carbenes were designed, synthesized, and studied for their ability to generate reactive oxygen species and target antioxidant pathways *via* multiple mechanisms.

## 1. Introduction

The regulation of reactive oxygen species (ROS) within biological systems plays a vital role in the health and longevity of many organisms.^1^,^2^ In disease states, such as cancer, the basal levels of ROS are elevated due to increased cellular growth combined with reduced waste elimination.^3^ Cellular adaptation to ROS in pre-neoplastic cells exposed to inducers (*i.e.*, hypoxia, metabolic defects, ER stress, oncogene activation, *etc.*) often result in increased antioxidant pathway activity.^4^ Biological systems have a host of natural defenses to ROS, including enzymes such as superoxide dismutase (SOD), glutathione peroxidase, and catalase. Additional cofactors, such as glutathione (GSH), thioredoxin (Trx)/thioredoxin reductase (TrxR), ascorbate (vitamin C), and α-tocopherol (vitamin E), are also able to serve as ROS scavengers.

Although cancer cells can thrive in the oxidative environment that they create, their ability to buffer ROS has limits. Both tumor and normal cells are driven to apoptosis when ROS levels become too high.^5^ This oxidative-stress pathway to apoptosis, if exploited, could be a new cancer treatment option.^4^ While any compound that disrupts redox homeostasis will negatively affect all cells, normal cells are thought to have a greater capacity for adaptation.^4^ Thus, it is expected that an agent that acts to increase oxidative stress will overload the capabilities of neoplastic cells, while being relatively less lethal to normal cells.

The chemotherapeutic development of agents that alter the redox environment within cancer cells have been categorized into (1) ROS generators (*e.g.*, motexafin gadolinium (MGd), β-lapachone, *etc.*) and (2) antioxidant system inhibitors (*e.g.*, buthionine sulphoximine, tetrathiomolybdate).^6^–^14^ Collectively, data gleaned from this work have provided insight into the cellular antioxidant system and has resulted in the proposal of oxidative stress modulation as an anticancer strategy.^4^,^15^

**Fig. 1:**
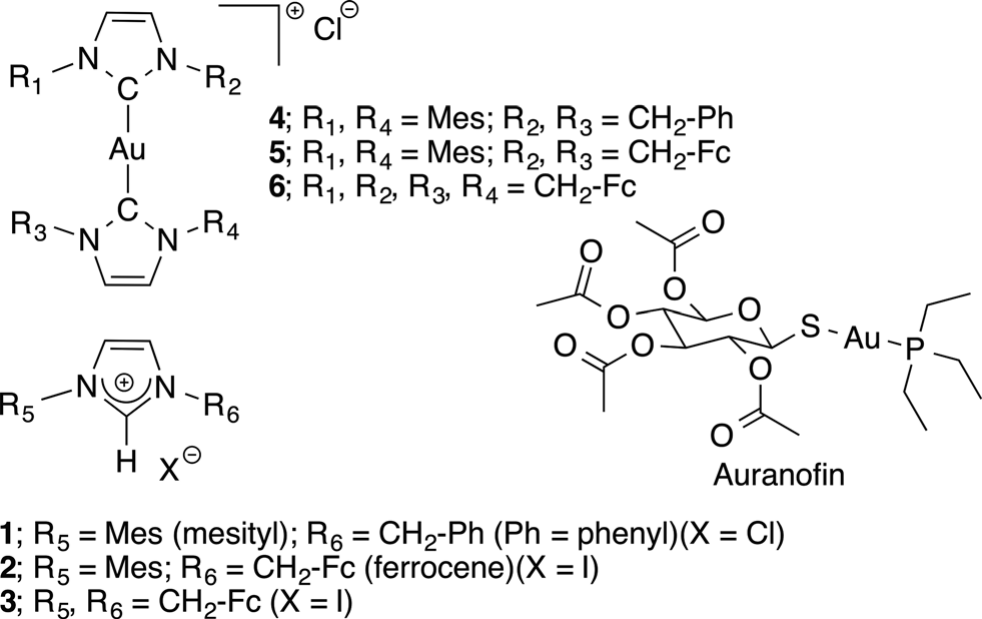
Structures of ferrocene containing imidazolium salts (**1–3**, previous work), their corresponding [Au(NHC)_2_]^+^ complexes (**4–6**, this work), and auranofin.

Within the antioxidant system, Trx plays a central role in mediating cellular response to environmental stress making the inhibition of Trx/TrxR an attractive strategy for patients undergoing radiation therapy.^16^,^17^ Studies with auranofin (see Fig. 1) revealed the ability of Au(i)-based compounds to inhibit TrxR *via* binding to the selenylsulfide/selenothiol redox center of the enzyme.^18^–^22^ This discovery resulted in the subsequent development of NHC ligated Au(i) complexes as anticancer therapeutic agents.^23^–^30^

While targeting antioxidant systems is a viable strategy for anticancer development, there are limited examples of complexes capable of affecting the cellular antioxidant system *via* multiple mechanisms.^31^–^37^ To explore the possibility of multiple modes of pathway targeting, we sought to develop a complex capable of (1) TrxR inhibition and (2) redox cycling, both of which would affect intracellular levels of ROS. Previous reports of ferrocene-containing gold(i) complexes have indicated activity against screened cancer cell lines; however, no mechanistic investigations have been reported.^38^

It is known that ferrocene (Fc) complexes elevate ROS within cancer cells and such complexes have become an increasingly popular motif in the development of therapeutics for treating cancer.^39^–^48^ One key feature of ferrocene is that it is capable of undergoing a one electron oxidation to form the corresponding ferrocenium cation (Fc^2+^ → Fc^3+^), a process that precedes the reductive regeneration often implicated in its cytotoxicity.^41^,^49^,^50^ These observations led us to hypothesize that a hybrid compound capable of inducing non-specific ROS (*via* ferrocene) in addition to selectively inhibiting TrxR (*via* binding to Au(i)) could override the ROS regulatory pathway in tumor models.^51^ To test this hypothesis, compounds **4–6** (*cf.*, Fig. 1), which contain (1) an Au(i) carbene core to inhibit TrxR and (2) ferrocene units designed to increase intracellular ROS levels were prepared and tested *in vitro* against the A549, A2780, 2780CP, and PC-3 cell lines. The underlying mechanism of action was also explored.

## 2. Experimental

### 2.1 Materials and methods

The following compounds were prepared according to literature procedures: 1-mesitylimidazole,^52^ [(Mes)(C_3_H_3_N_2_)(CH_2_C_6_H_5_)][Cl] (Mes = mesityl),^53^,^54^ 1-(ferrocenylmethyl)-3-mesitylimidazolium iodide,^55^ 1,3-di(ferrocenylmethyl)imidazolium iodide,^56^ and (C_4_H_2_S)AuCl.^57^ All other reagents were purchased from commercial sources and used as received, including: [((CH_3_)_3_Si)_2_N]Na (NaHMDS) and [(Cp_2_Fe)CH_2_N(CH_3_)_3_][I] (Cp = cyclopentadienyl). CDCl_3_, CD_2_Cl_2_, and DMSO-*d*_6_ (99.9%) were purchased from Cambridge Isotope Laboratories, dried over 3 Å molecular sieves, and degassed using three consecutive freeze–pump–thaw cycles prior to use. Solvents were either dried with a solvent purification system from the Vacuum Atmosphere Company (CH_2_Cl_2_, Et_2_O, hexanes and toluene) or freshly distilled over 3 Å molecular sieves and degassed using three consecutive freeze–pump–thaw cycles prior to use. All reactions and manipulations were conducted under an atmosphere of nitrogen unless otherwise indicated. UV-vis spectra were obtained at ambient temperature with a Hewlett-Packard 8452A diode array spectrophotometer. Molar absorptivities are reported in M^–1^ cm^–1^. ^1^H and ^13^C NMR spectra were recorded on JEOL 400 MHz spectrometer. Spectra were referenced to the residual solvent as an internal standard, for ^1^H NMR: CD_2_Cl_2_, 5.32 ppm; CDCl_3_, 7.24 ppm; DMSO-*d*_6_, 2.50 ppm; for ^13^C NMR: CD_2_Cl_2_, 54.00 ppm; CDCl_3_, 77.0 ppm, and DMSO-*d*_6_, 39.5 ppm. Coupling constants (*J*) are expressed in hertz (Hz). High-resolution mass spectra (HRMS) were obtained using a VG analytical ZAB2-E or a Karatos MS9 instrument (ESI or CI) and are reported as *m*/*z* (relative intensity). Electrochemical measurements were performed on a CHI660D or Pine Wavenow electrochemical workstation using a silver wire quasi-reference electrode, a platinum disk working electrode, and a Pt wire auxiliary electrode in a gas tight three-electrode cell under an atmosphere of nitrogen. Unless specified otherwise, the measurements were performed using 1.0 mM solutions of the analyte in dry DMSO with 0.1 M [N(*n*Bu)_4_][PF_6_] as the electrolyte and decamethylferrocene (Fc*) as the internal standard. Differential pulse voltammetry measurements were performed with 50 mV pulse amplitudes and 2 mV data intervals. All potentials listed herein were determined by cyclic voltammetry at 100 mV s^–1^ scan rates and referenced to a saturated calomel electrode (SCE) by shifting decamethylferrocene^0/+^ to –0.030 V (DMSO).^58^,^59^ Elemental analyses were performed by Midwest Microlab, LLC in Indianapolis, IN. Cell culture media consisted of RPMI 1640 with 2 mM glutamine and 25 mM HEPES (Corning 10041CV) with 10% heat-inactivated fetal bovine serum (Sigma f6178) and 1X penicillin-streptomycin (Sigma p4333). Trypsin (Hyclone SH30236.01) and Dulbecco's Phosphate Buffer Saline (Sigma d8537) were used for general cell maintenance and harvesting. Cell lines were obtained from the ATCC (A549 and PC-3) and Prof. Zahid Siddik at MD Anderson (A2780 and A2780CP). Thiazolyl Blue tetrazolium bromide (Alfa Aesar L11939) was used for cell proliferation assays. Cell culture plastic ware consisted of generic T-75 flasks, 80.5 mm diameter culture dishes, and treated 96-well plates.

### 2.2 Syntheses

#### Bis(1-benzyl-3-mesitylimidazol-2-ylidene)-gold(i) chloride (**4**)

An 8 mL screw cap vial equipped with a stir bar was charged with [Mes(C_3_H_3_N_2_)CH_2_Ph]^+^[Cl]^–^ (100 mg, 0.320 mmol) and NaN(SiMe_3_)_2_ (64.5 mg, 0.035 mmol). Dry toluene (2 mL) was added to the vial and the resulting mixture was stirred at 25 °C for 30 min. The reaction mixture was then filtered through a plug of Celite into a 8 mL vial containing a slurry of (C_4_H_8_S)AuCl (46.2 mg, 0.144 mmol) in 1 mL of dry toluene. The resulting mixture was stirred at 25 °C for 30 min. After the volatiles were removed under reduced pressure, the resulting crude product was dissolved in 2.5 mL of dichloromethane and filtered through a plug of Celite into a 20 mL vial. The filtrate was then treated with activated charcoal to remove colored impurities. After filtering through a plug of Celite and trituration with hexanes (12 mL), a white precipitate was obtained. The precipitate was subjected to series of washes (3 × 3 mL of hexanes and then 2 × 3 mL of diethyl ether) to yield a white solid. The resulting crude product was recrystallized from dichloromethane/diethyl ether to give colorless crystals. Yield: 43%. ^1^H NMR (δ, CDCl_3_, 400 MHz): 1.79 (s, 12H, Mes-CH3), 2.23 (s, 6H, Mes-CH_3_), 5.17 (s, 4H, CH_2_), 6.82 (br s, 4H, Mes), 6.88 (br s, 2H, PhNCH), 7.13 (m, 4H, Ph), 7.28 (m, 6H, Ph), 7.60 (s, 2H, MesNCH). ^13^C NMR (δ, CDCl_3_, 125 MHz): 17.67, 21.16, 54.35, 122.92, 123.10, 127.51, 128.48, 128.94, 129.21, 134.87, 135.99, 139.63, 184.18. HRMS (ESI) for [C_38_H_40_N_4_Au]^+^ [M^+^] calcd 749.2919 found 749.2927. Anal. calcd for: C_38_H_40_N_4_AuCl: C, 58.13; H, 5.13; N, 7.14; found: C, 57.88; H, 5.32; N, 7.01.

#### Bis(1-(ferrocenylmethyl)-3-mesitylimidazol-2-ylidene)-gold(i) chloride (**5**)

An 8 mL screw cap vial equipped with a stir bar was charged with [Fe(η^5^-C_5_H_4_CH_2_(C_3_H_3_N_2_)(Mes))Cp]^+^[I]^–^ (100 mg, 0.196 mmol) and NaN(SiMe_3_)_2_ (43.3 mg, 0.236 mmol). Dry toluene (2 mL) was added to the vial and the resulting mixture was stirred at 25 °C for 30 min. The reaction mixture was then filtered through a plug of Celite into an 8 mL vial containing a slurry of (C_4_H_8_S)AuCl (28.2 mg, 0.088 mmol) in 1 mL of dry toluene. The resulting mixture was then stirred at 25 °C for 20 min. After the volatiles were removed under reduced pressure, the resulting crude product was dissolved with 2.5 mL of dichloromethane and then filtered through a plug of Celite into a 20 mL vial. The filtrate was then treated with activated charcoal to remove colored impurities. After filtration and trituration with hexanes (12 mL), a yellow precipitate was obtained. The yellow precipitate was subjected to series of washes (3 × 3 mL of hexanes and then 2 × 3 mL of diethyl ether) to yield a yellow solid. Yield: 65%. ^1^H NMR (δ, CDCl_3_, 400 MHz): 1.76 (s, 12H, Mes), 2.34 (s, 6H, Mes), 4.14 (s, 4H, Fc), 4.16 (s, 10H, Fc), 4.17 (s, 4H, Fc), 5.08 (s, 4H, CH2), 6.82 (s, 2H, FcNCH), 6.89 (s, 4H, Mes), 7.69 (s, 2H, MesNCH). ^13^C NMR (δ, CDCl_3_, 125 MHz): 17.74, 21.33, 50.77, 68.65, 68.94, 69.05, 82.58, 122.47, 122.75, 129.27, 134.81, 134.88, 139.45, 183.20. HRMS (ESI) for C_46_H_48_N_4_Fe_2_Au [M]^+^ calcd 965.2243, found 965.2253. Anal. calcd for: C_46_H_48_N_4_Fe_2_AuCl: C, 55.19; H, 4.83; N, 5.60; found: C, 54.40; H, 5.62; N, 5.51.

#### Bis(1,3-di(ferrocenylmethyl)imidazol-2-ylidene)-gold(i) chloride (**6**)

An 8 mL vial equipped with a stir bar was charged with [Fe(η^5^-C_5_H_4_CH_2_(C_3_H_3_N_2_)(CH_2_-η^5^-C_5_H_4_FeCp))Cp]^+^[I]^–^ (100 mg, 0.170 mmol) and NaN(SiMe_3_)_2_ (38.7 mg, 0.211 mmol). Dry toluene (2 mL) was added to the vial and the resulting mixture was stirred at 25 °C for 90 min. The reaction mixture was then filtered through a plug of Celite into an 8 mL vial containing a slurry of (C_4_H_8_S)AuCl (24.5 mg, 0.0764 mmol) in 1 mL of dry toluene. The resulting mixture was then stirred at 25 °C for 20 min. After the volatiles were removed under reduced pressure, the resulting crude product was dissolved with 5 mL of dichloromethane and filtered through a plug of Celite into a 20 mL vial. The filtrate was then treated with activated charcoal to remove colored impurities. After filtration and trituration with hexanes (12 mL), a yellow precipitate was obtained. The resulting precipitate was subjected to series of washes (3 × 3 mL hexanes and then 2 × 3 mL diethyl ether), yielding a yellow solid. The product was recrystallized from a mixture of dichloromethane/methanol by treating with pentane, which produced reddish brown crystals. Yield: 64%. ^1^H NMR (δ, CDCl_3_ and CD_3_OD, 400 MHz): 4.18 (s, 20H, Fc), 4.22 (br s, 8H, Fc), 4.31 (s, 8H, Fc), 5.12 (s, 8H, CH_2_), 7.13 (s, 4H, N(CH)_2_N). ^13^C NMR (δ, CDCl_3_ and CD_3_OD, 125 MHz): 51.21, 69.02, 69.18, 82.06, 121.29, 182.13. HRMS [M]^+^ for [C_50_H_48_N_4_Fe_4_Au]^+^, calcd 1125.0942, found 1125.0951. Anal. calcd for: [C_50_H_48_N_4_Fe_4_Au][Cl_0.5_I_0.5_]: C, 49.78; H, 4.01; N, 4.64. Found: C, 49.77; H, 4.73; N, 4.65.

### 2.3 X-ray crystallography

Golden yellow crystals of **5** were obtained by diffusing diethyl ether into a CH_2_Cl_2_ solution. Diffusing methyl *tert*-butyl ether into a 1,2-dichloroethane solution provided bright yellow crystals of **6**. Data for **5** and **6** were collected on a Rigaku AFC12 diffractometer with a Saturn 724+ CCD using a graphite monochromator with MoKα radiation (*λ* = 0.71073 Å) equipped with a Rigaku XStream cooling system (100 K). Data were collected using 1 degree omega scans for both **5** and **6**. For **5**, 1560 frames were collected at 30 seconds per frame, while for **6**, 1320 frames were collected at 40 seconds per frame. Data were collected under control of the Rigaku Americas Corporation's Crystal Clear version 1.40 (Rigaku Americas Corporation, 2008). Structure solutions were obtained by direct methods for all compounds using SIR 2004.^60^,^61^ Refinements were accomplished by full-matrix least-squares procedures using the SHELXL-2014 (G. M. Sheldrick, SHELXL/PC package (version 5.1), program for the refinement of crystal structures, University of Gottingen, 2003).^62^,^63^ In many instances, the cyclopentadienyl rings on the ferrocenyl units displayed rotational disorder that was generally treated with distance and angle constraints. Rigid bond restraint was used in some instances to treat atoms attached to the gold(i) center because some of the carbon atoms bound to the gold(i) atom went non-positive definite. All hydrogen atoms were added in calculated positions and included as riding contributions with isotropic displacement parameters tied to those of the atoms to which they were attached. Additional crystallographic details may be found in the respective CIFs, which were deposited at the Cambridge Crystallographic Data Centre (CCDC), Cambridge, UK. For CCDC numbers, please refer to the ESI.†

### 2.4 *In vitro* anti-proliferative activity

The proliferation of exponential phase cultures of A549 cells was assessed by tetrazolium salt reduction. In brief, tumor cells were seeded in 96-well microliter plates at 1000 cells per well and allowed to adhere overnight in 100 μL RPMI 1640 medium supplemented with 2 mM l-glutamine, 10% heat-inactivated fetal bovine serum, and antibiotics (200 U cm^–3^ penicillin and 200 μg cm^–3^ streptomycin). Stock solutions of complex (10 mM in DMSO or 5 mM in 50/50 v/v water/DMSO) were formulated and then diluted in medium for secondary stocks of 100–200 μM depending on the complex being tested. Secondary stock solutions were serially diluted in medium and immediately added to wells, whereupon plates were incubated at 37 °C under a 5% CO_2_/95% air atmosphere. After a total of three days, a 50 μL aliquot of 3 mg mL^–1^ tetrazolium dye, 3-(4,5-dimethylthiazol-2-yl)-2,5-diphenyltetrazolium bromide (MTT, Sigma Chemical Co.), was added to each well, followed by a four hour incubation period at 37 °C. The medium was then removed, the resulting formazan was dissolved in 50 μL DMSO and the respective absorbances were measured at 560–650 nm using a microplate reader (Molecular Devices, Sunnyvale, CA). Absorbance values were corrected for background and then normalized to wells containing untreated cells to allow for plate-to-plate comparisons. The data are shown as mean inhibition of proliferation or growth as a percentage of control cells and are from 2–3 replicate experiments.

### 2.5 ICP-MS determination of Fe and Au

To determine complex uptake in tumor cell lines, ICP-MS studies were undertaken using an A549 lung cancer cell line. Cells were seeded in 150 cm^2^ cell culture flasks and grown to confluence in 30 mL RPMI 1640 medium supplemented with 2 mM l-glutamine, 10% heat inactivated fetal bovine serum, and antibiotics (200 U cm^–3^ penicillin and 200 μg cm^–3^ streptomycin). The media was removed and supplemented with 30 mL of media (with FBS and antibiotics) containing 2.5 μM of complex originating from 2.5 mM stock complex in DMSO. The cells were then allowed to incubate at 37 °C under a 5% CO_2_/95% air atmosphere for 6 h. The medium from each sample was then removed, and cells were washed with PBS (made in-house with ICP-MS grade deionized water), treated with trypsin, and pelleted in 15 mL conical tubes. The pellets were washed 2× with 10 mL of PBS and the cells were counted with a hemocytometer. Cell counts consisted of 25–40 million cells per sample. Samples were pelleted, frozen over dry ice, digested with conc. HNO_3_, and analyzed by Applied Analytical, Inc. for total Fe and Au content.

### 2.6 Determination of relative ROS levels through FACS analysis

Tumor cells (2–3 × 10^6^) were plated overnight and then incubated with media containing one of the complexes described above at concentrations of 2.5 μM. Control cells were treated with vehicle only. At defined time-points, the media was collected and the cells were washed with PBS. The PBS washing was collected and the attached cells were treated with trypsin and collected. The loosened cells were passed through a 40 μm cell strainer. All media and washings were collected, pelleted by centrifugation (3 min @ 300 g) and washed twice with cold PBS. The cells were once again pelleted and suspended in PBS at a final concentration of 2 × 10^6^ cells per mL. To each of the 15 mL centrifuge tubes was added 100 μL of the cell suspension before being incubated in the dark at 37 °C for 15 min at a final concentration of 1 mg mL^–1^ CM-DCFA. PBS (2 mL) was added to each sample. The cells were then pelleted, washed 2× with PBS, and re-suspended in 5 μg mL^–1^ of propidium iodide (PI) in PBS. Control samples of unstained cells, cells stained with only CM-DCFA, and cells stained with only PI were also prepared. Each sample was added to one well of a 96-well plate. Samples were then subjected to FACS analysis using a Millipore Guava easyCyte 8 and analyzed using the Guava inCyte software.

### 2.7 Lipoate reduction

To 96-well plates containing plateau phase A549 lung cells was added 2.5 μM of each complex described above in RPMI-16 media containing 10% FBS and P/S. The cells were left to incubate for 6 h at 37 °C at 5% CO_2_. At this point, the media was removed and replaced with HBSS buffer containing 20 mM lipoate and 1 mM 5,5′-dithiobis-2-nitrobenzoic acid (DTNB). The plates were immediately monitored at 405 nm in a time dependent fashion. Time points were collected every 20 min and the *T*_0_ value was subtracted from each point.

### 2.8 Determination of intracellular Zn levels through FACS analysis

The concentration of intracellular free zinc was assessed using the ion-specific fluorescent probe, FluoZin-3-AM (FluoZin-3, Molecular Probes, Inc.) Plateau phase cultures grown in T-25 flasks were treated with control vehicle or zinc acetate in the presence or absence of 2.5 μM of a complex as described above for 4 h. After treatment, the cells were washed with PBS and treated with trypsin/EDTA for 5 min. Complete medium was then added and the cells were isolated by centrifugation. Cell pellets were washed and re-suspended in PBS. An aliquot of 1 × 10^6^ cells was removed, centrifuged, and re-suspended in 100 μL of 20 μmol L^–1^ FluoZin-3 in PBS. After a 25 min incubation period under ambient conditions, the cells were washed twice with PBS, re-suspended in 0.5 mL PBS, and then incubated for 20 min. An aliquot of the cell suspension was supplemented with 2 μg mL^–1^ propidium iodide (Sigma), incubated for 5 min, and subjected to two-parameter flow cytometric analysis.

### 2.9 Isolation of RNA for microarray analysis

Ribonucleic acid was isolated for microarray analysis using the QIAGEN RNeasy Plus Universal Mini Total RNA extraction protocol. About 5 000 000 cells of A549 were seeded into each cell culture dish (Corning 430293) with 11 mL RPMI 1640 with 2 mM l-glutamine, 10% heat-inactivated fetal bovine serum, 200 U cm^–3^ penicillin, and 200 μg cm^–3^ streptomycin. Each pair of dishes was seeded from the same T-75 flask. After being incubated overnight at 37 °C with 5% CO_2_, another 11 mL of the previously-described media were added to control dishes, while treated dishes received 11 mL of **6** at a concentration of 5 μM in the previously-described media, for a final concentration of 2.5 μM. After incubating at 37 °C with 5% CO_2_ for 6 h, the media was removed by aspiration. The cells were washed with warm PBS and then treated with trypsin. The trypsin was quenched using complete medium and the cells were transferred into centrifuge tubes. The cells were centrifuged for 5 min at 1000 rpm in an Eppendorf 5804 centrifuge. The supernatant was removed and cells were re-suspended in 380 μL PBS. Each sample was then treated with 900 μL QIAzol Lysis Reagent, and homogenized by vigorous vortexing and shaking. The QIAGEN RNeasy Plus Universal Mini Total RNA protocol was then picked up at Step 4. RNA was eluted in two volumes of 30 μL each for a final volume of 60 μL RNA. The centrifuges used include an Eppendorf 5804R for steps performed at 4 °C and an Eppendorf MiniSpin Plus for steps performed at room-temperature. RNA was stored at –80 °C. The RNA concentration was measured using a Thermo Scientific NanoDrop 2000c Spectrophotometer. A gel of 1% agarose with in TAE was cast and RNA was run at 125 V for 60 min to ensure the integrity of the RNA through visualization of ribosomal subunits. The ladder used was the Thermo SM 1331 Generuler 1 kb + dsDNA ladder. Each sample (400 ng + 3 μL) was submitted for microarray analysis to the Interdisciplinary Center for Biotechnology Research at the University of Florida.

## 3. Results and discussion

### 3.1 Syntheses and characterization

**Fig. 2:**
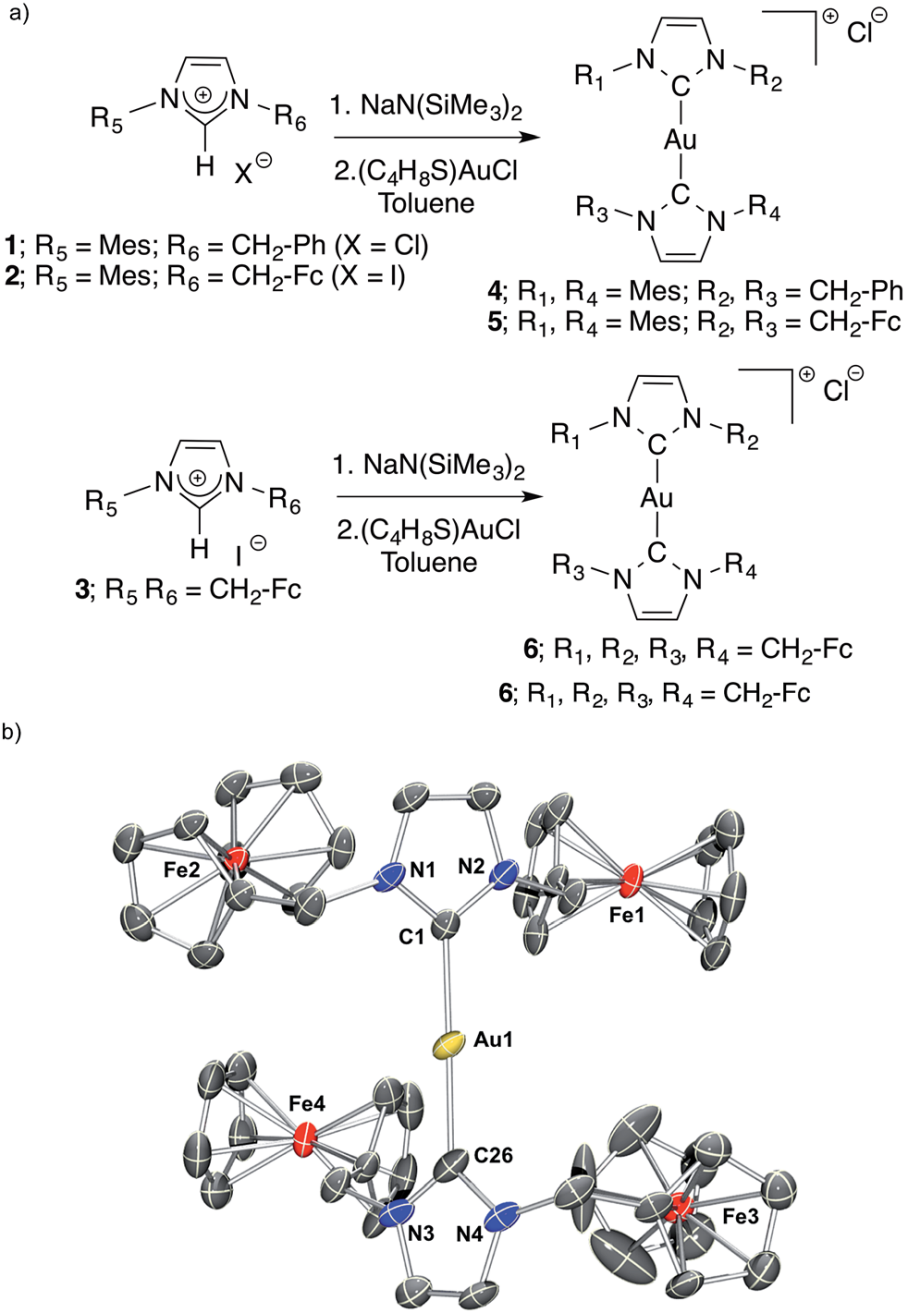
(a) Synthesis of ferrocene containing Au(i) carbene complexes. (b) ORTEP diagram of **6** rendered using POV-Ray. Thermal ellipsoid plots are drawn at the 50% probability level. Hydrogen atoms and counter anion are omitted for clarity. Selected bond lengths (Å) and angles (deg): C1–N1, 1.37(1); C1–N2, 1.35(2); C1–Au1, 2.021(9); C26–Au1, 2.028(8); C26–N3, 1.33(1); C26–N4, 1.35(1); N1–C1–N2, 105.2(8); N3–C26–N4, 105.1(7); C1–Au1–C26, 176.5(3).

As shown in Fig. 2, compounds **4**, **5**, and **6** were synthesized using a modified literature procedure by independently treating free carbenes generated *in situ* with (C_4_H_8_S)AuCl.^64^ The resulting complexes were isolated as microcrystals after titration of the corresponding saturated CH_2_Cl_2_ solutions with *n*-hexanes, followed by series of washes with *n*-hexanes and diethyl ether. The complexes were subjected to a variety of characterization techniques, including ^1^H NMR, ^13^C NMR and ultraviolet-visible spectroscopy. The appearance of diagnostic ^13^C NMR signals (C_carbene_) at 184.18 ppm (CDCl_3_), 183.20 ppm (CDCl_3_) and 182.13 ppm (CDCl_3_ and CD_3_OD) for **4**, **5** and **6**, respectively, were consistent with the values reported for analogous Au–N-heterocyclic carbene (NHC) complexes.^38^,^65^ Compounds **5** and **6** displayed a dipole-forbidden absorption band around 440 nm and a shoulder at 528 nm, consistent with the values reported in the literature for analogous ferrocene containing species (see the ESI†). The aforementioned absorption bands were absent for compound **4**, consistent with the aforementioned assignment. Single crystals of **5** suitable for X-ray diffraction analysis were grown by slowly diffusing diethyl ether into a concentrated CH_2_Cl_2_ solution. Similarly, single crystals of **6** were grown by slowly diffusing methyl *tert*-butyl ether into a concentrated 1,2-dichloroethane solution. Thermal ellipsoid plots of **5** and **6** are presented in Fig. S1† and 2b, respectively. Compound **6** crystallizes in the triclinic space group *P*1, while compound **5** crystallizes in the monoclinic space group *C*2/*c*. Compounds **5** and **6** adopted a linear geometry with C–Au–C bond angle ∼177°. The N–C–N and C–Au–C bond angles were in accordance with data reported for other analogous [Au(NHC)_2_]^+^ gold(i) complexes.^38^,^65^ All Au–C_carbene_ bonds distances were in the range 2.00 (7)–2.03 (1) Å.

To elucidate the electronic properties of compounds **2**, **3**, **5**, and **6**, a series of electrochemical measurements were carried out in DMSO with [N(*n*Bu_4_)][PF_6_] as the electrolyte; key data are summarized in Table 1. Compounds **2**, **3**, **5**, and **6** all displayed an iron centered (Fe^2+^ → Fe^3+^) reversible oxidations. One electron oxidations for **2** and **3** was observed at ∼0.59 V (*vs.* SCE), whereas the relatively electron rich [Au(NHC)_2_]^+^ complexes underwent oxidation at ∼0.56 V. No gold oxidation was observed under the experimental conditions employed.

**Table 1:** Electrochemical data recorded for various ferrocenylated complexes^*a*^

Compound	*E*_1/2_ ^*a*^ (V)
**2**	0.58 (r)
**3**	0.59 (r)
**5**	0.56 (r)
**6**	0.57 (r)

^*a*^The potentials shown were obtained *via* differential pulse voltammetry measurements in DMSO with 0.1 M [N(*n*Bu)_4_]^+^[PF_6_]^–^ electrolyte, 0.1 mM analyte, and referenced *vs.* SCE. See the ESI for the corresponding cyclic voltammograms and differential pulse voltammograms. r = reversible.

### 3.2 Ability to inhibit cell proliferation

**Fig. 3:**
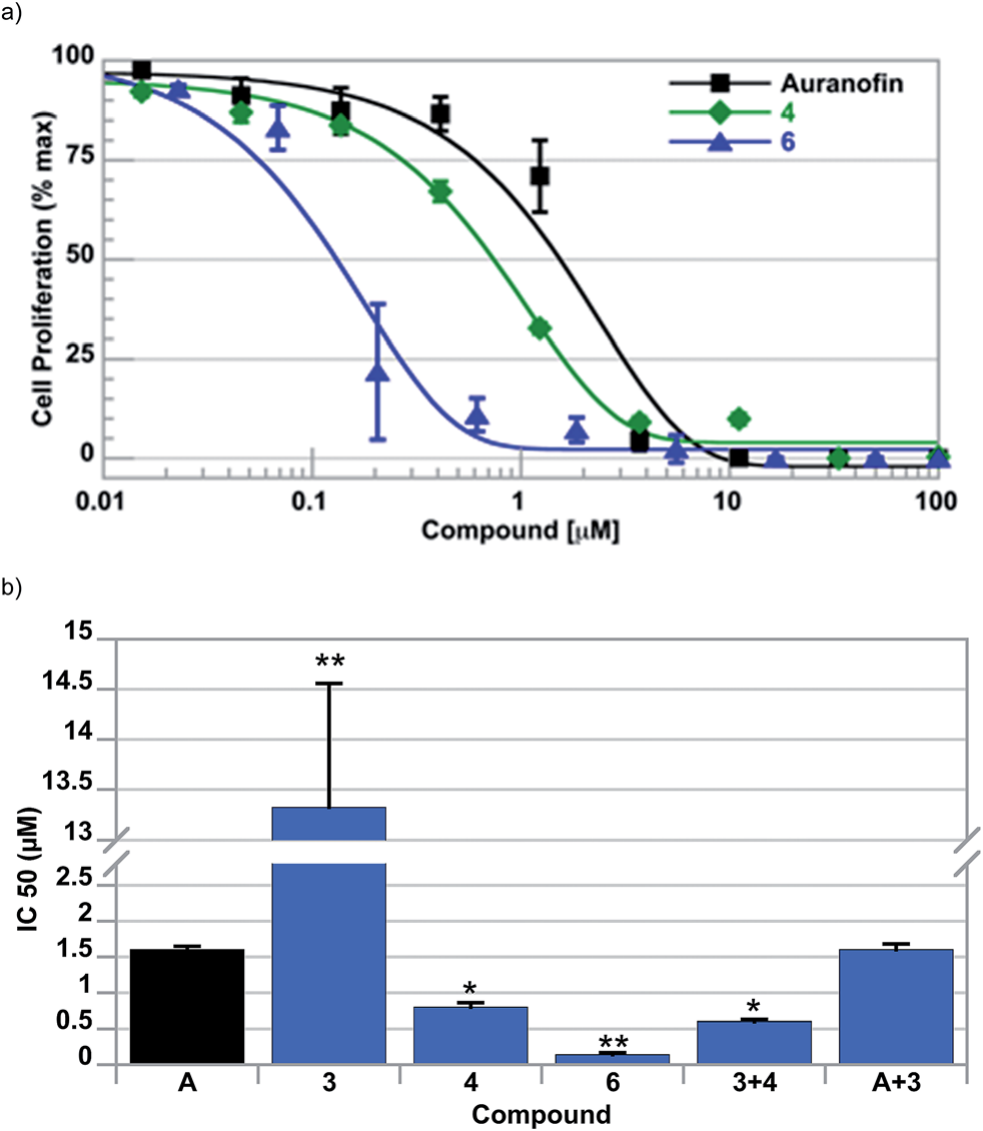
(a) Cell proliferation profiles and (b) bar graph plot summarizes the potency of all the compounds explored. Note that the potency of **6** is greater than the 2 : 1 mixture of compounds **3** and **4**. Error bars represent one standard deviation. A one-way ANOVA with Dunnet's *post-hoc* test was used to compare each compound with auranofin (*P < 0.05; **P < 0.01).

To assess the ability, if any, of the individual complexes of this study to inhibit cell growth, cell proliferation assays were conducted following exposure of A549 lung cells to **2–6** and a control compound, auranofin; key data are summarized in Table 2. Typical dose–response curves were observed with all complexes investigated (*cf.*Fig. 3a). It was observed that potency was directly proportional to amount of ferrocene contained within the complex (*i.e.*, IC_50_ of **6** < **5** < **4**). The potency of **6** (IC_50_ = 0.14 ± 0.03 μM) was found to be >10 fold greater than auranofin (IC_50_ = 1.67 ± 0.05 μM) in this cell line. In addition, it was found that the Au-containing complexes displayed significantly greater potency (>100-fold) than the individual ferrocene subunits (*i.e.*, compounds **2** and **3**). To assess the contribution of each moiety of **6** to the observed cell proliferation inhibitory effects, A549 cells were exposed to variable concentrations of **3** + **4** and auranofin + **3**, both in a 1 : 2 molar ratio, and compared to **6**. A combined dose of [Au(NHC)_2_]^+^**4** and ferrocene **3** provided a slight synergistic effect (*i.e.* IC_50_ = 0.61 ± 0.05 μM *vs.* 0.71 ± 0.03 μM), while mixtures of auranofin and **3** provided no improvement. Regardless, the ability of **6** to inhibit cell proliferation was still significantly greater than the sum of its constituent parts (c.f. Fig. 3b).

**Table 2:** IC_50_ values for compounds tested on A549 lung cancer cells

Complex	IC_50_ (μM)^*a*^	Complex	IC_50_ (μM)
**2**	6.4 ± 1.1	**6**	0.14 ± 0.03
**3**	13 ± 1.5	Auranofin (A)	1.67 ± 0.05
**4**	0.71 ± 0.03	**3** + **4**	0.61 ± 0.05
**5**	0.39 ± 0.01	Auranofin (A) + **3**	1.61 ± 0.09

^*a*^Standard deviation is noted (3–5 repeat runs).

Previous studies had indicated an increase in the potency of ferrocenium relative to ferrocene.^26^ To test if this relationship was relevant to the complexes described above, **5** was oxidized to [**5**][BF_4_]_2_ and examined for its ability to inhibit cell proliferation of A549 lung cells. The oxidized ferrocenium compound ([**5**][BF_4_]_2_) was tested in conjunction with **5** and no difference was observed in its ability to inhibit A549 cell growth (see ESI†).

Complex **6** was further screened with PC-3 prostate (p53 null), A2780 (wt-p53 platinum sensitive), and 2780CP (wt-p53 isogenic partner to A2780 displaying multidrug resistance (MDR)) (see Table 3).^66^,^67^ Inspection of the IC_50_ values indicated similar potencies across all cell lines with no observed resistance in 2780CP relative to A2780 cell lines.

**Table 3:** IC_50_ values for compound **6** in various cancer cell lines

Cell line	A549 lung	A2780 ovarian	2780CP ovarian	PC-3 prostate
IC_50_ (μM)^*a*^	0.14 ± 0.03	0.19 ± 0.01^*c*^	0.12 ± 0.01^*c*^	0.48 ± 0.15^*b*^

^*a*^Standard deviation is noted (3–5 repeat runs).

^*b*^A Dunnet's *post-hoc* test revealed that the IC_50_ for compound **6** was only different in PC-3 cells.

^*c*^Tukey's test was used to verify that the potency of compound **6** was equal in the A2780 and A2780CP cell lines.

### 3.3 Assessing cellular uptake *via* ICP-MS

**Fig. 4:**
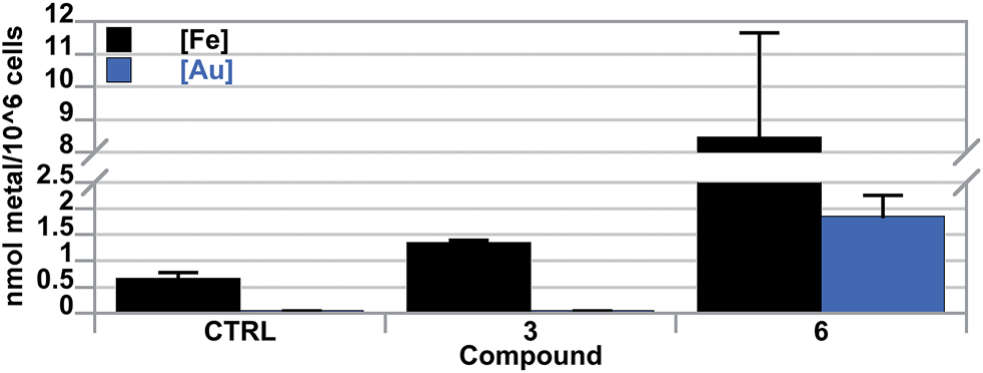
ICP-MS detection of intracellular Fe and Au levels from A549 cells treated with compounds **3** and **6**. Error bars represent one standard deviation. Although a biological trend is evident, statistical analysis (*i.e.*, one-way ANOVA) resulted in a *P* value of 0.09. This result may be explained by our small sample size (*N* = 2).

To assess complex integrity and uptake, inductively coupled plasma mass spectrometry (ICP-MS) studies were carried out with the goal of quantifying Fe and Au levels. In brief, A549 cells were independently exposed to 2.5 μM of **6** or **3** for 6 h. Cells were then collected, counted, and quantified for intracellular uptake of Fe and Au *via* ICP-MS (*cf.*Fig. 4). An increase in Fe was evident in both samples treated with **6** as well as **3**. Relative to **3**, Fe was quantified as 11-fold higher in cells after exposure to **6**. This result suggested to us that cellular uptake of **6** was 5.6-fold higher than that of **3** in A549 lung cancer cells and is consistent with the ∼100-fold difference observed in the ability of **6** to inhibit cell proliferation. Gold was also detected in cells exposed to **6**. Subsequent analysis resulted in a 4 : 1 ratio of Fe : Au in cells exposed to **6**, indicative that the [Au(NHC)_2_]^+^ complex is stable and enters the cell as a whole complex.

### 3.4 Reactive oxygen species disruption

It is proposed that the combination of ferrocene moieties and Au–NHC complexes present in the compounds of this study results in a system that is capable of disrupting ROS regulation *via* multiple mechanisms. To assess the ability of the aforementioned complexes to disrupt and increase ROS levels, fluorescence assisted cell-sorting (FACS) analyses were conducted utilizing 5-(and-6)-chloromethyl-2′,7′-dichlorodihydrofluorescein diacetate, acetyl ester (CM-H_2_DCFDA), a fluorescein based indicator for general ROS fluctuations. Due to the relatively high potencies of the [Au(NHC)_2_]^+^ complexes studied, low drug incubation concentrations were needed to avoid cellular stress. A549 cells were thus exposed to variable concentrations of complex **5** for 4 hours, collected and treated with propidium iodide (PI) to assess cytotoxicity. It was found that a concentration of 2.5 μM of [Au(NHC)_2_]^+^ was sufficient to allow for exposure without killing cells within the 4 hour incubation period (see the ESI†).

**Fig. 5:**
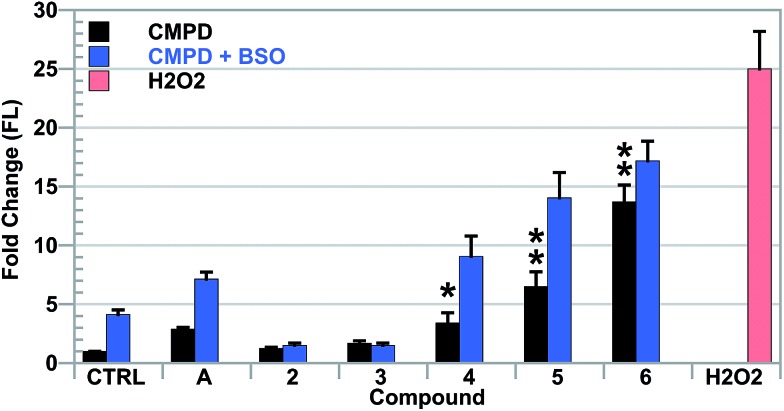
Reactive oxygen species detected by fluorescent signal increases of DCF *via* flow cytometric analysis in live A549 cells treated with various complexes. H_2_O_2_ was used as a positive control. Error bars represent one standard deviation. A one-way ANOVA with Dunnet's *post-hoc* test was used to compare each compound with the vehicle control (*P < 0.05; **P < 0.01).

Complexes **4**, **5**, **6** and auranofin were independently added to A549 cells and their ability to increase ROS was examined (*cf.*Fig. 5). It was found that while all of the complexes induced an ROS increase relative to control samples, complexes **4**, **5**, and **6** provided higher levels than that observed when auranofin was utilized. The greatest increase in ROS was detected in cells exposed to complex **6**, which provided a 14-fold increase in ROS relative to cells treated with vehicle.

As previously mentioned, l-buthionine-(*S*,*R*)-sulfoximine (BSO) acts as a selective inhibitor of GSH synthesis.^25^,^68^,^69^ The down regulation of GSH, a ROS scavenger, could potentiate the effects of the gold complexes of this study, thus providing support for the proposed mode of action.^70^ To test this hypothesis, A549 cells were exposed to BSO for a 24 h period before being independently treated with [Au(NHC)_2_]^+^ complexes **4**, **5**, **6** or auranofin (*cf.*Fig. 5). It was observed that cells with reduced levels of GSH provided increased levels of ROS upon exposure to the various Au(i)-containing complexes of this study.

### 3.5 Inhibition of thioredoxin reductase

**Fig. 6:**
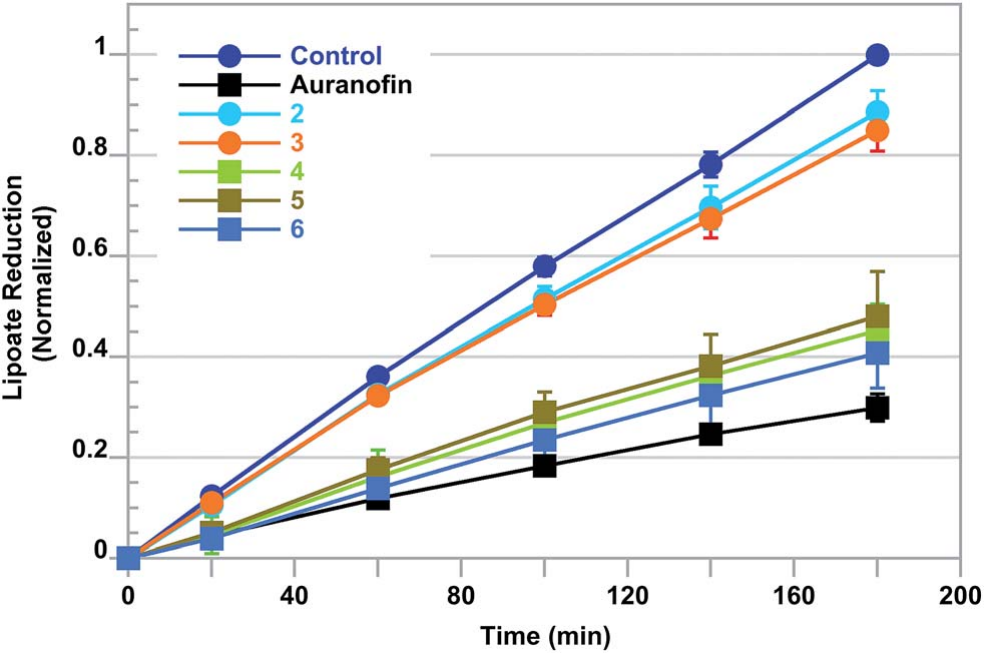
Time-dependent inhibition of thioredoxin reductase (TrxR) *via* the reduction of lipoate. Error bars represent standard deviation. For clarity, statistical symbols were not included in this figure. A one-way ANOVA with Dunnet's *post-hoc* test was used to compare TrxR activity at the 180 minute time point. At this time point, all compounds were statistically different from the control (*P* < 0.05: **2** and **3**; *P* < 0.001: auranofin, **4**, **5**, and **6**) and auranofin (*P* < 0.05: **5** and **6**; *P* < 0.001: **2**, **3**, and **4**) in their ability to inhibit TrxR.

It has previously been reported that auranofin and [Au(NHC)_2_]^+^ are able to bind to and inhibit TrxR, a feature considered integral to their mode of cytotoxic action.^23^–^26^ This literature suggestion, combined with our findings that l-buthionine-(*S*,*R*)-sulfoximine (BSO) treatments (a GSH inhibitor) sensitizes A549 cells towards increases in ROS production when independently exposed to **4**, **5**, or **6**, led us to investigate whether these complexes would also have an effect on the thioredoxin pathway. The live cell measurement of thioredoxin reductase activity may be accomplished by monitoring the reduction of the oxidized form of the cell-permeable cofactor lipoate to its reduced form, dihydrolipoate. To this effect, the plateau phase A549 cells were independently exposed to 2.5 μM treatments of [Au(NHC)_2_]^+^**4–6**, auranofin and **2** or **3** for 6 h. After treatment, the cells were then monitored colorimetrically for their ability to reduce lipoate (*cf.*Fig. 6). It was observed that the A549 cells treated with auranofin provided a 70% reduction in TrxR activity relative to samples treated with vehicle. Thioredoxin reductase inhibition in cells independently treated with [Au(NHC)_2_]^+^ complexes **4–6** was found to also be significant and ranged from 55–60% inhibition. A more modest (*i.e.*, 10–15%) inhibition was observed in cells treated with **2** or **3**. These results were taken as evidence that complexes **4–6** are capable of TrxR inhibition similar to that of auranofin (a positive control). This mechanism is thought to contribute, in part, to the potency observed in complexes containing both [Au(NHC)_2_]^+^ and ferrocene.

### 3.6 Intracellular free zinc elevation

**Fig. 7:**
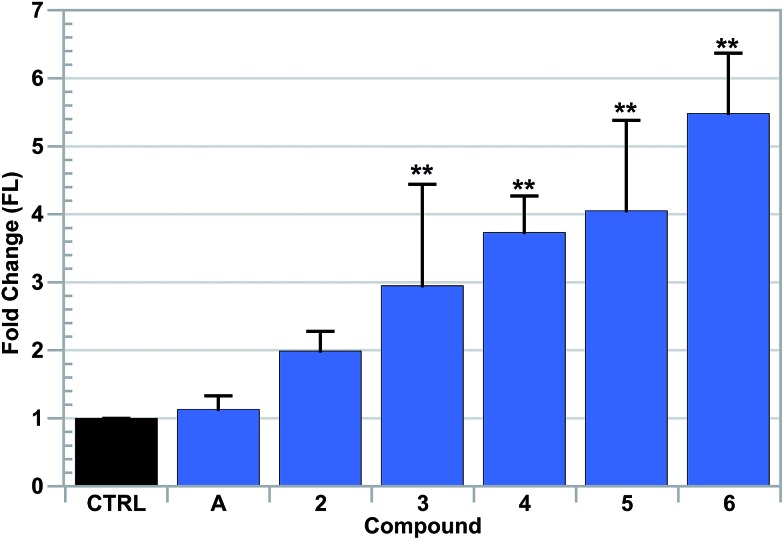
Detection of intracellular zinc fluctuations by fluorescent signal increases of FluoZin-3 *via* flow cytometric analysis in live A549 cells treated with various complexes. Error bars represent one standard deviation. A one-way ANOVA with Dunnet's *post-hoc* test was used to compare each compound with the vehicle control (**P < 0.01).

The above findings suggested to us that the complexes **4–6** can weaken the antioxidant system in different ways. ROS increases could affect reducing species containing vicinal thiols bound to zinc, such as metallothionein. This, in turn, would produce intracellular zinc as an additional by-product of redox cycling. To test this hypothesis, cultures of A549 cells were independently incubated with [Au(NHC)_2_]^+^ complexes **4–6**, auranofin, or **2** or **3** for 6 hours. At that point, FACS analysis was used to detect (chelatable) intracellular zinc with the ion-specific dye, FluoZin-3 (*cf.*Fig. 7).^71^ It was found that complexes **4–6** induced the greatest amount of zinc increase, with **6** providing the most substantial elevation in free zinc (*i.e.*, a 5-fold increase). A moderate 2–3 fold increase in Zn was detected in live cells treated with **2** or **3**, while no Zn increase was detected in samples treated with auranofin. Upon co-treatment with 100 μM zinc acetate, a significant increase (*i.e.* 45-fold) in intracellular zinc was detected in cells treated with complexes **4–6**, whereas little to no change was detected in auranofin, **2**, or **3** (see the ESI†). In addition, upon further study, little to no synergistic effect between **6** and Zn was detected in cell proliferation assays (see the ESI†).

### 3.7 Gene expression in treated A549 cells

To assess the effects of the complexes on gene expression profiles, total cellular RNA was isolated from plateau phase A549 cultures treated with 2.5 μM complex **6** for 6 h in triplicate and analyzed on RNA microarrays.^72^,^73^ These conditions were chosen based on the consideration that no cell death by **6** was observed within 6 h of treatment. All 740 transcripts (including control and non-coding genes) that were differentially expressed (up-regulation: >1.4-fold, down-regulation: <0.7-fold, corrected *P* < 0.15) in response to treatment with **6** are presented (see the ESI†). For clarity, a list of coding transcripts that were most differentially expressed is listed in Table 4. As one might anticipate, transcripts with cell death/growth/survival-related functions (*e.g.*, *DDIT3*, *SESN2*, and *GDF15*) were identified. The list also includes transcripts involved in endoplasmic reticulum stress/response to stress (*i.e. CHAC1*, *DDIT3*, *TRIB3*, *ASNS*, *etc.*), HIF-1 (*i.e.*, *HMOX1*), zinc transport (*SLC30A1*), metallothioneins (MT; five metallothionein related transcripts), and heat shock transcription factors (*HSF*; *e.g.*, four heat shock-related transcripts).

**Table 4:** RNA microarray analysis: differential expression of select genes in A549 cells treated with **6**

Gene ID	Gene symbol	Gene description	FC	*P*-value
79094	*CHAC1*	ChaC, cation transport regulator homolog 1	5.56	1.19205 × 10^–7^
1649	*DDIT3*	DNA-damage-inducible transcript 3	4.43	4.30297 × 10^–9^
57761	*TRIB3*	Tribbles pseudokinase 3 (TRIB3)	4.41	6.5417 × 10^–8^
440	*ASNS*	Asparagine synthetase (glutamine-hydrolyzing)	3.94	3.28201 × 10^–8^
27063	*ANKRD1*	Ankyrin repeat domain 1 (cardiac muscle)	3.90	0.000102275
9518	*GDF15*	growth differentiation factor 15	2.90	9.19533 × 10^–6^
7779	*SLC30A1*	Solute carrier family 30 (zinc transporter)	2.86	0.002992119
23645	*PPP1R15A*	Protein phosphatase 1, regulatory subunit 15A	2.79	7.03697 × 10^–7^
7436	*VLDLR*	Very low density lipoprotein receptor	2.56	3.7443 × 10^–6^
9709	*HERPUD1*	Homocysteine-inducible, endoplasmic reticulum stress-inducible, ubiquitin-like domain	2.48	2.53382 × 10^–7^
2081	*ERN1*	Endoplasmic reticulum to nucleus signaling 1	2.44	1.02427 × 10^–6^
3162	*HMOX1*	Heme oxygenase (decycling) 1	2.41	6.63288 × 10^–5^
4495	*MT1G*	Metallothionein 1G	2.24	0.034444693
467	*ATF3*	Activating transcription factor 3	2.10	1.231 × 10^–6^
16	*AARS*	Alanyl-tRNA synthetase	2.03	3.39778 × 10^–6^
2920	*CXCL2*	Chemokine (C-X-C motif) ligand 2	1.93	0.00014337
4490	*MT1B*	Metallothionein 1B	1.92	0.032952383
3576	*CXCL8*	Chemokine (C-X-C motif) ligand 8	1.92	1.10178 × 10^–5^
7494	*XBP1*	X-box binding protein 1	1.87	3.60972 × 10^–5^
4496	*MT1H*	Metallothionein 1H	1.77	0.025658976
3311	*HSPA7*	Heat shock 70 kDa protein 7	1.63	0.008645638
6782	*HSPA13*	Heat shock protein 70 kDa family, member 13	1.54	0.001471724
3309	*HSPA5*	Heat shock 70 kDa protein 5 (glucose-regulated protein, 78 kDa)	1.51	2.83009 × 10^–5^
29948	*OSGIN1*	Oxidative stress induced growth inhibitor 1	1.48	0.000144965
57181	*SLC39A10*	Solute carrier family 39 (zinc transporter), member 10	0.63	0.016893383
3306	*HSPA2*	Heat shock 70 kDa protein 2	0.56	1.49155 × 10^–5^
6347	*CCL2*	Chemokine (C-C motif)	0.39	1.53404 × 10^–5^

## 4. Discussion

A series of ferrocene containing [Au(NHC)_2_]^+^ complexes were designed as models for dual targeting of specific pathways. The ferrocene and [Au(NHC)_2_]^+^ moieties were specifically chosen to (1) generate intracellular ROS non-selectively and (2) selectively inhibit TrxR, an enzyme essential in the ROS response pathway. We chose to explore this binary approach as a novel way to target and overwhelm specific pathways. Disruption of ROS regulatory systems is attractive in the context of drug design due to the fact that cancers typically display elevated ROS levels.^15^ While cancer selectivity (*i.e.*, healthy tissue *vs.* tumors) has yet to be established in the present instance, it is evident that there is a positive correlation between ROS generation and the ability of a drug candidate to inhibit cell proliferation. We were able to successfully show an increase in ROS generation that was both positively correlated with the number of ferrocene subunits incorporated in the complex, and an ability to inhibit cell proliferation (*i.e.*, for **6**, IC_50_ = 0.14 μM, 13.7-fold increase of intracellular ROS). This is a significant increase in potency relative to the control compound auranofin (IC_50_ = 1.67 μM, 2.7-fold increase in ROS) whose primary mode of action involves TrxR inhibition.^18^–^20^,^22^–^26^ Downregulation of GSH by pre-treatment with BSO was found to potentiate the effects of all complexes, thus providing support for the proposed mode of action.^70^

A key finding to emerge from this study is that the potency of **6** is greater than the sum of its parts (*i.e.*, its antiproliferative activity that is greater than that of **3** + **4**). It was experimentally confirmed *via* ICP-MS analyses that the intracellular uptake of **6** was greater than that of **3**, and may reflect increased ferrocene delivery through the action of the [Au(NHC)_2_]^+^ complex. The difference in uptake between **6** and **3** could also reflect the altered amphiphilicity of **6** relative to **3**. It may be inferred that the reduced potency of **3** (or any combination of complexes that include **3**) may be due to poor cellular uptake and not a lack of ferrocene activity. However, a key point is that the potency of complex **6** is multifactorial and cannot be accounted for solely in terms of the number of ferrocene units it contains.

As would be expected in light of the proposed dual targeting mode of action, the present [Au(NHC)_2_]^+^ complexes were found to inhibit TrxR. Complexes **4**, **5**, and **6** were found to inhibit ∼55% of TrxR activity, while auranofin inhibited activity by 70%. This difference in inhibition may be due to differences in the coordination chemistry of [Au(NHC)_2_]^+^ carbene (*i.e.*, **6**) and Au(i)–phosphine complexes (*i.e.* auranofin). The significant increase in ROS and inhibition of TrxR by the ferrocenylated [Au(NHC)_2_]^+^ complexes was further corroborated by an intracellular increase in free zinc (also indicative of ROS increase/stress response).

RNA microarray gene expression was used to elucidate further the mechanism of **6**. Of the 279 genes that were differentially expressed, a significant number were associated with apoptosis and cell cycle arrest, as might be expected. Gene Ontology (GO) analyses of the transcripts that were differentially-regulated in response to exposure to compound **6** were performed to investigate cellular responses to this complex. Intriguingly, ER stress and oxidative stress response genes were found to be enriched in this analysis (see Table 4 and S2†). These data, coupled with the differential expression of *HMOX1* (containing an antioxidant response element in its promoter) and *OSGIN1* (an oxidative response protein that regulates cell death), were taken as an indication that the oxidative stress induced by **6** results in ER stress. The subsequent upregulation of *SLC30A1*, downregulation of *SLC39A10* (both Zn transporters) and upregulation of multiple metallothioneins are thought to reflect a response to ROS stress since they serve to attenuate an increase in intracellular zinc concentrations. The role intracellular free (non-protein bound) zinc plays in regulating cellular functions is of considerable relevance to cancer. For example, increased free zinc concentration has been proposed to stabilize hypoxia-inducible factor-1 (HIF-1) and thus influence processes such as glycolysis, apoptosis, and angiogenesis.^74^–^77^ Moreover, free zinc inhibits thioredoxin reductase,^72^ a key mediator in the cellular response to oxidative stress that is frequently overexpressed in cancer.^78^–^80^

The scope of activity of gold complex **6** was further evaluated within a limited panel of cancer cell lines PC3 prostate (p53 null), A2780 ovarian (wt-p53 platinum sensitive), and 2780CP (wt-p53 isogenic partner to A2780 displaying multidrug resistance (MDR)) displaying varying p53 status and drug resistance. From these results, it should be noted that there was no observed resistance in 2780CP relative to A2780 cell lines. This result is considered significant in that small molecular platinum containing species often display 2–27 fold resistance between this isogenic pair.^66^,^67^

## 5. Conclusions

Herein we report that ferrocenylated N-heterocyclic carbene supported Au(i) complexes are capable of targeting antioxidant pathways by regulating ROS *via* multiple mechanisms. The proposed incorporation of ROS-generating ferrocenes on a Au(i) platform capable of TrxR inhibition provided complexes with enhanced anti-proliferative properties relative to ferrocene or Au(i) alone (*e.g.*, auranofin or **4**). It also provides initial “proof-of-principle” support for the suggestion that it is useful to address key cancer-related pathways *via* multiple modes of targeting. The utility of complex **6**, for example, in treating potential cross-resistance across a number of cell lines is also appealing. Accordingly, further mechanistic studies, tests of toxicity and efficacy in mammalian models, as well as efforts to prepare and test second-generation complexes that are able to accentuate ROS effects *via* multiple pathways are underway. The results of these efforts will be presented in due course.

## Supplementary Material

Supplementary information

Crystal structure data
