# Supplementary material for: Targeting antioxidant pathways with ferrocenylated N-heterocyclic carbene supported gold(i) complexes in A549 lung cancer cells
Source: Chem Sci. 2015 Oct 29;7(2):1245–56. doi: 10.1039/c5sc03519h (PMC4762604; doi:10.1039/c5sc03519h)
Supplement: Supplementary file 1 [file SC-007-C5SC03519H-s001.pdf]

Electronic Supplementary Material (ESI) for Chemical Science.
This journal is © The Royal Society of Chemistry 2015

Supporting Information

Targeting Antioxidant Pathways with Ferrocenylated *N*-Heterocyclic Carbene Supported Gold(I) Complexes in A549 Lung Cancer Cells

J. F. Arambula,[a]* R. McCall,[a] K. J. Sidoran,[b] D. Magda,[c] N. A. Mitchell,[d] C. W. Bielawski,[e,f] V. M. Lynch,[g] J. L. Sessler[g], and K. Arumugam[b]*

[a] Department of Chemistry, Georgia Southern University, Statesboro, Georgia, 30460, USA.
[b] Department of Chemistry, Wright State University, 3640 Colonel Glenn Hwy, Dayton, Ohio, 45435, USA.
[c] Lumiphore, Inc., Berkeley, California, 94710, USA.
[d] Department of Health Sciences, Gettysburg College, Gettysburg, PA 17325-1400
[e] Center for Multidimensional Carbon Materials, Institute for Basic Science, Ulsan 689-798, Republic of Korea.
[f] Department of Chemistry and Department of Energy Engineering, Ulsan National Institute of Science and Technology (UNIST), Ulsan 689-798, Republic of Korea
[g] Department of Chemistry, University of Texas at Austin, Austin, Texas, 78712, USA.

*E-mail: jarambula@georgiasouthern.edu; kuppuswamy.arumugam@wright.edu

Table of Contents

**Synthesis**	S2-S2
**X-ray Crystallography**	S2-S2
**Electrochemical Data**	S3-S4
**^{1}H and ^{13}C NMR Spectra**	S5-S10
**UV-Vis Absorption Spectra**	S11-S11
**Biological Assays**	S12-S13
**RNA Microarray Heat Map and Differential Gene Expression**	S14-S26
**References**	S26-S26

Synthesis

An 8 mL screw cap vial equipped with a stir bar was charged with compound **5** (40 mg, 0.039 mmol) and $AgBF_4$. (5.5 mg, 0.084 mmol). Dry CH_2Cl_2 was added to the vial and the resulting mixture was stirred at 25 °C for 4 h. During this time the bright yellow solution turned to a dark green solution, indicative of formation of ferrocenium species. At the end of 4 h stir, the reaction mixture was filtered through a plug of Celite into a 8 mL vial. The volatiles were removed under reduced pressure and the resulting crude product was washed with 3 × 2 mL Et_2O. The resulting product was dried under reduced pressure to yield a dark green solid. Yield: 83%. Refer **Figure S13** for electronic spectra.

Table S1. Crystallographic and refinement data.

	Compound **5**	Compound **6**
CCDC	1419940	1419941
solvent	CH_2Cl_2	none
formula	$C_{94}H_{100}N_8Au_2Fe_4Cl_6$	$C_{100}H_{96}N_8Au_2Fe_8I_2$
fw	2171.85	1252.2
xtl system	monoclinic	triclinic
space grp	*C*2/*c*	*P*-1
color, habit	yellow, block	yellow, block
a, Å	27.578(1)	12.387(2)
b, Å	11.919(1)	12.624(2)
c, Å	27.111(1)	15.234(3)
α, deg.	90.00	90.566(5)
β, deg.	95.0850(8)	98.317(4)
γ, deg.	90.00	114.321(4)
V, Å^3	8876.55(42)	2138.99(60)
T, K	100(2)	100(2)
Z	4	2
R1, wR2[a]	0.049, 0.129	0.064, 0.183
GoF on F^2	1.204	1.049

[a] $R1 = \Sigma||F_o| - |F_c||/\Sigma|F_o|$. [b] $R_w = \{[\Sigma w(F_o^2 - F_c^2)/\Sigma w(F_o^2)^2\}^{1/2}$; $w = 1/[\sigma^2(F_o^2) + (xP)^2]$, where $P = (F_o^2 + 2F_c^2)/3$.

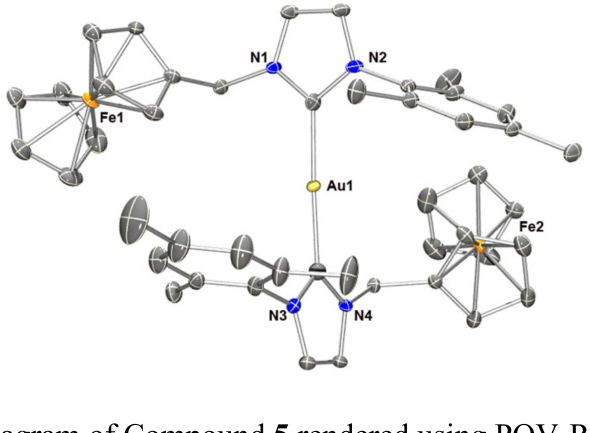

Figure S1. ORTEP diagram of Compound **5** rendered using POV-Ray. Thermal ellipsoid plots are drawn at the 50% probability level. Hydrogen atoms and counter anion are omitted for clarity. Selected bond lengths (Å) and angles (deg): C1–N1, 1.346(9); C1–N2, 1.353(9); C1–Au1, 2.000(7); C24–Au1, 2.023(6); C24–N3, 1.346(8), C24–N4, 1.339(8); N1–C1–N2, 104.6(6), N3–C24–N4, 105.8(5); C1–Au1–C24, 177.1(3).

Electrochemistry

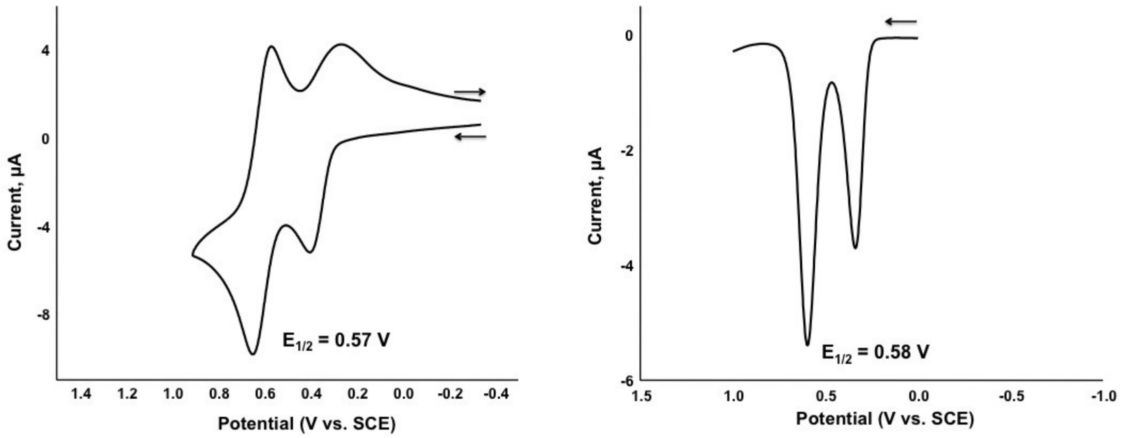

Figure S2. CV (100 mV s^{-1} scan rate) and DPV (50 mV pulse amplitude) of compound **2** in DMSO (1 mM) and 0.1 M $[N(n\mathrm{Bu}_4)]^+[PF_6]^-$ as referenced to decamethylferrocene (Fc*) (internal standard, adjusted to −0.030 V vs. SCE).[1]

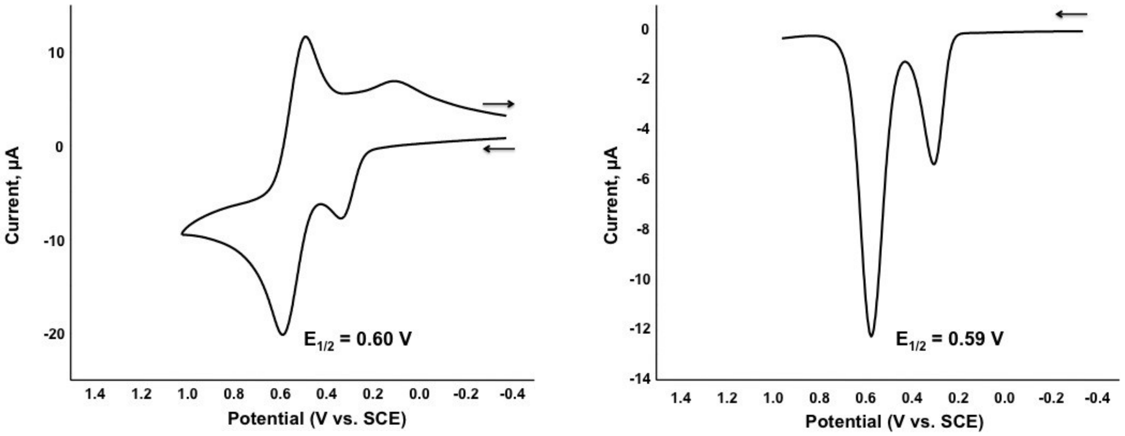

Figure S3. CV (100 mV s^{-1} scan rate) and DPV (50 mV pulse amplitude) of compound **3** in DMSO (1 mM) and 0.1 M $[N(n\mathrm{Bu}_4)]^+[PF_6]^-$ as referenced to decamethylferrocene (Fc*) (internal standard, adjusted to −0.030 V vs. SCE).[1]

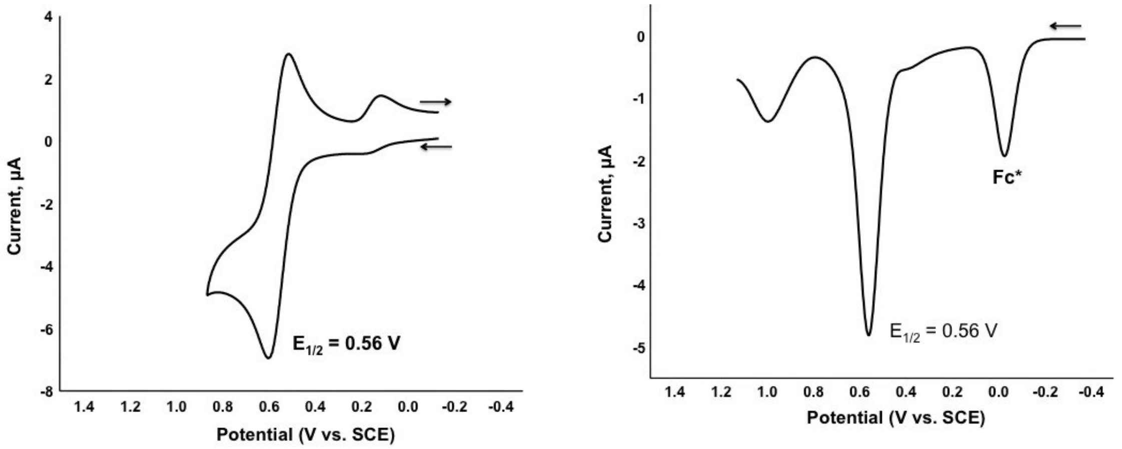

Figure S4. CV (100 mV s^{-1} scan rate) and DPV (50 mV pulse amplitude) of compound **5** in DMSO (1 mM) and 0.1 M $[N(nBu_4)]^+[PF_6]^-$ as referenced to decamethylferrocene (Fc*) (internal standard, adjusted to −0.030 V vs. SCE).[1]

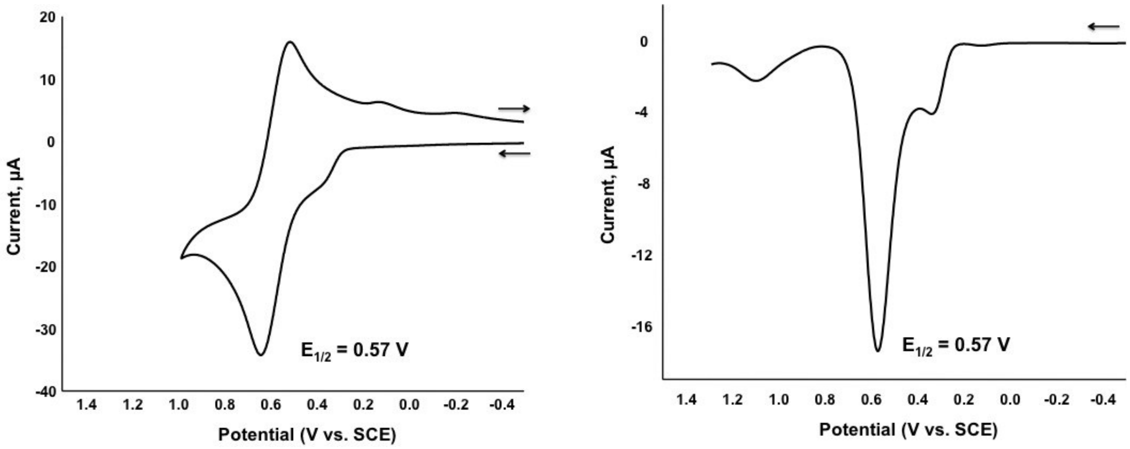

Figure S5. CV (100 mV s^{-1} scan rate) and DPV (50 mV pulse amplitude) of compound **6** in DMSO (1 mM) and 0.1 M $[N(nBu_4)]^+[PF_6]^-$ as referenced to decamethylferrocene (Fc*) (internal standard, adjusted to −0.030 V vs. SCE).[1]

^{1}H and ^{13}C NMR Spectra

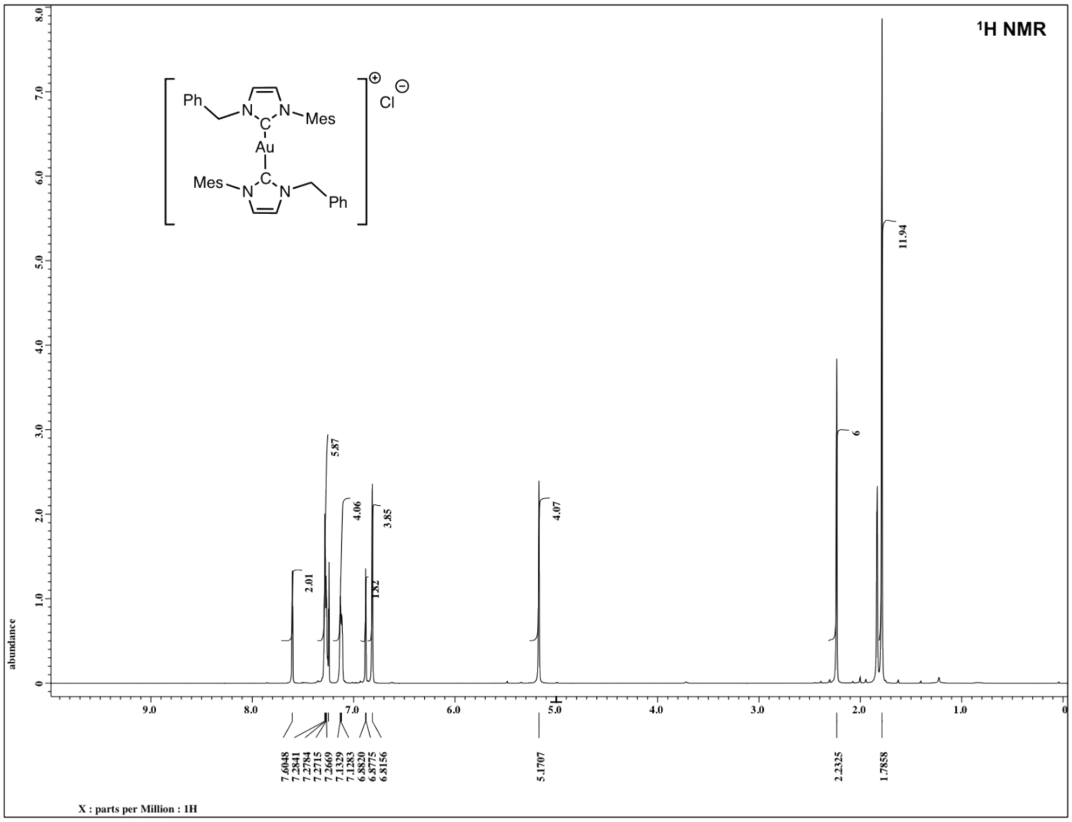

Figure S6. ^{1}H NMR spectrum of compound **4**.

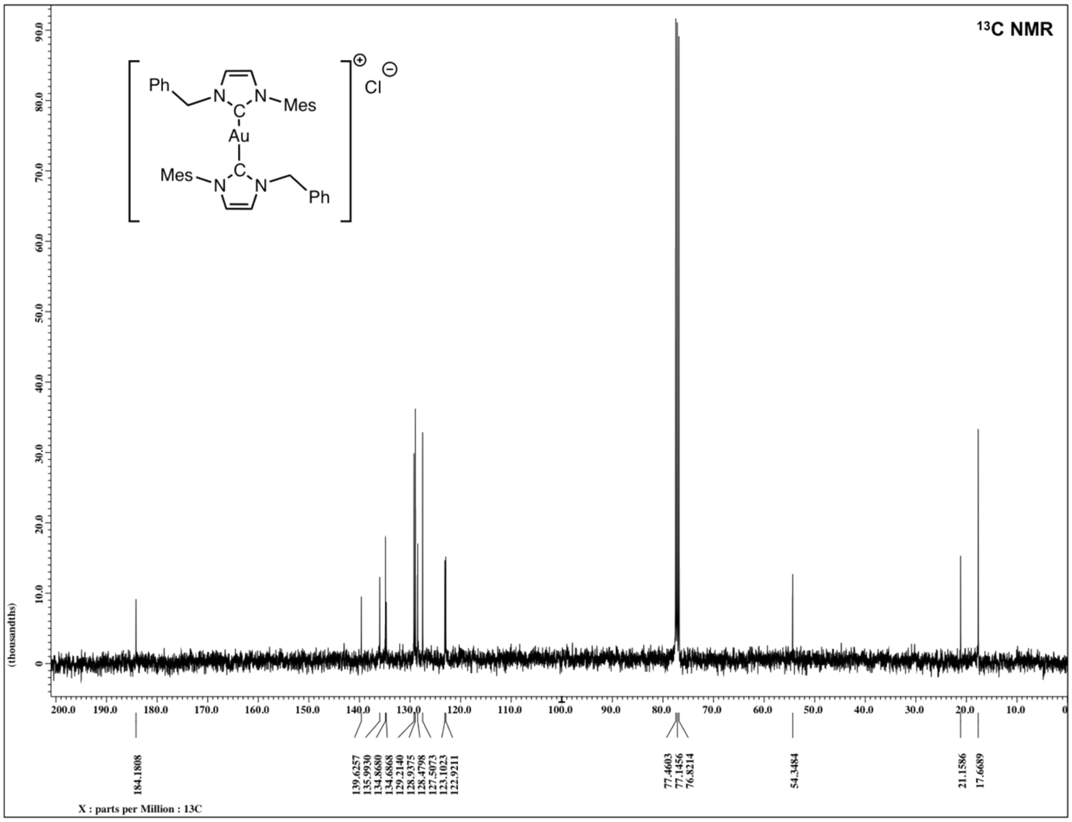

Figure S7. ^{13}C NMR spectrum of compound **4**.

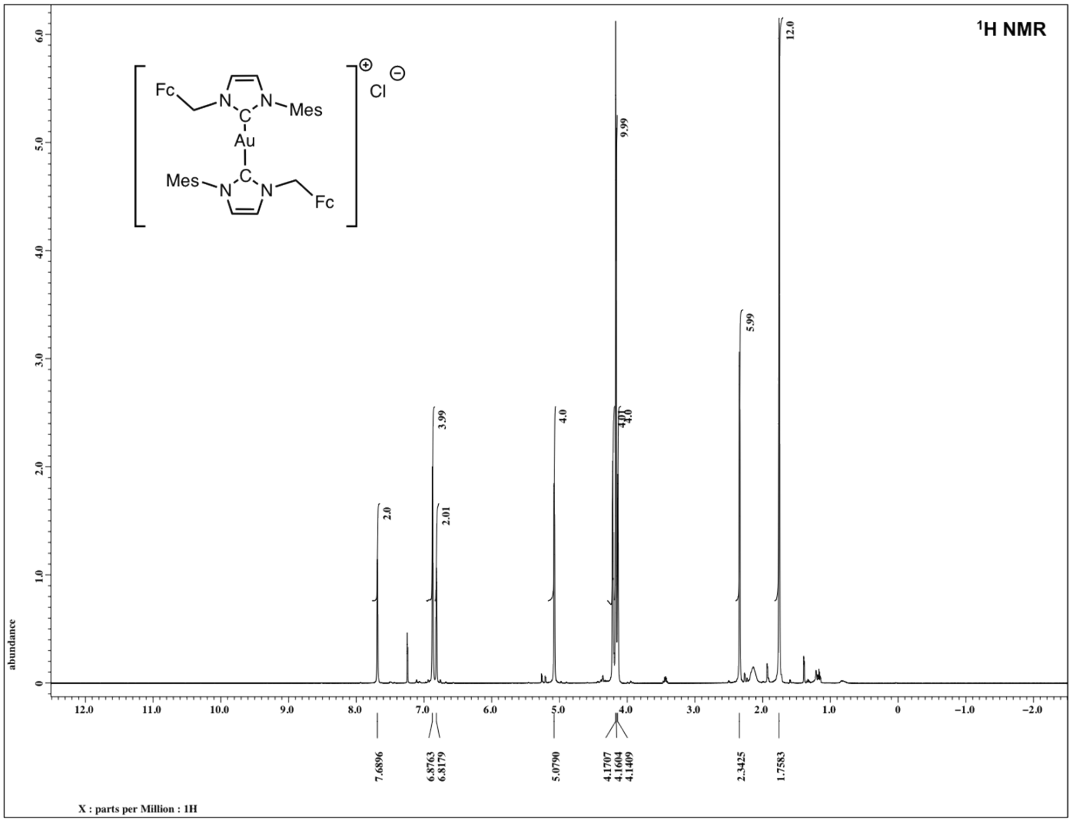

Figure S8. ^{1}H NMR spectrum of compound **5**.

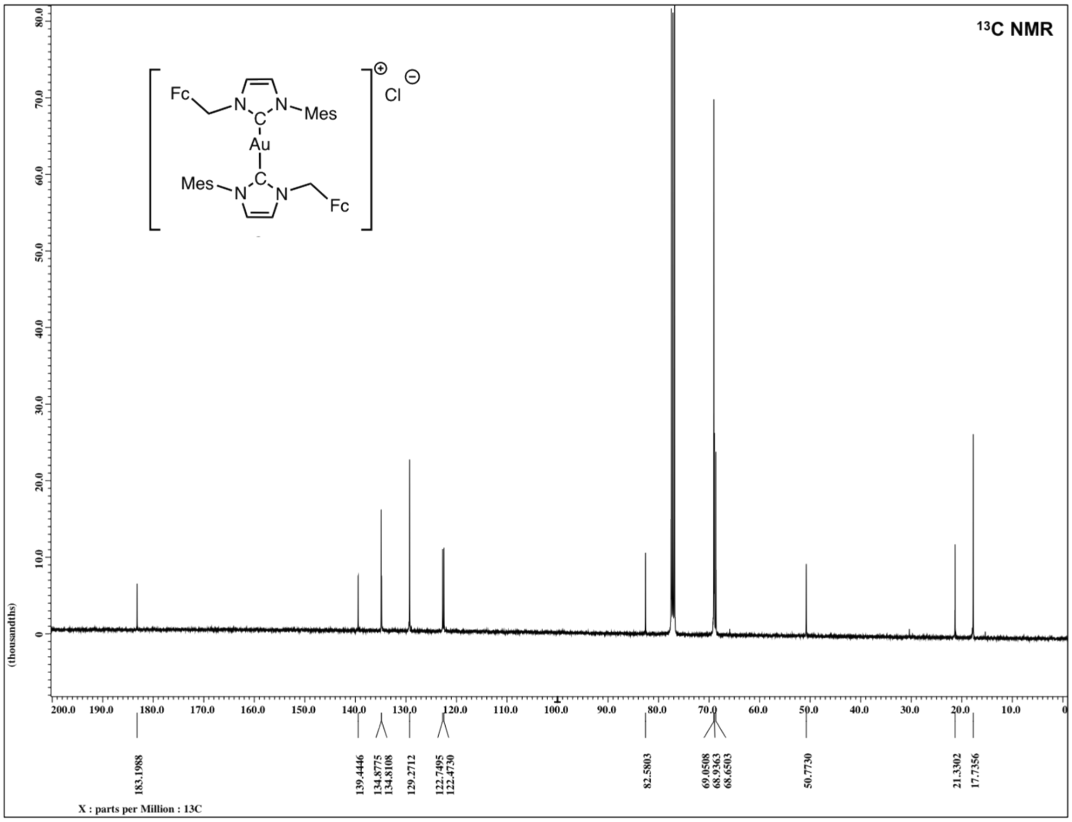

Figure S9. ^{13}C NMR spectrum of compound **5**.

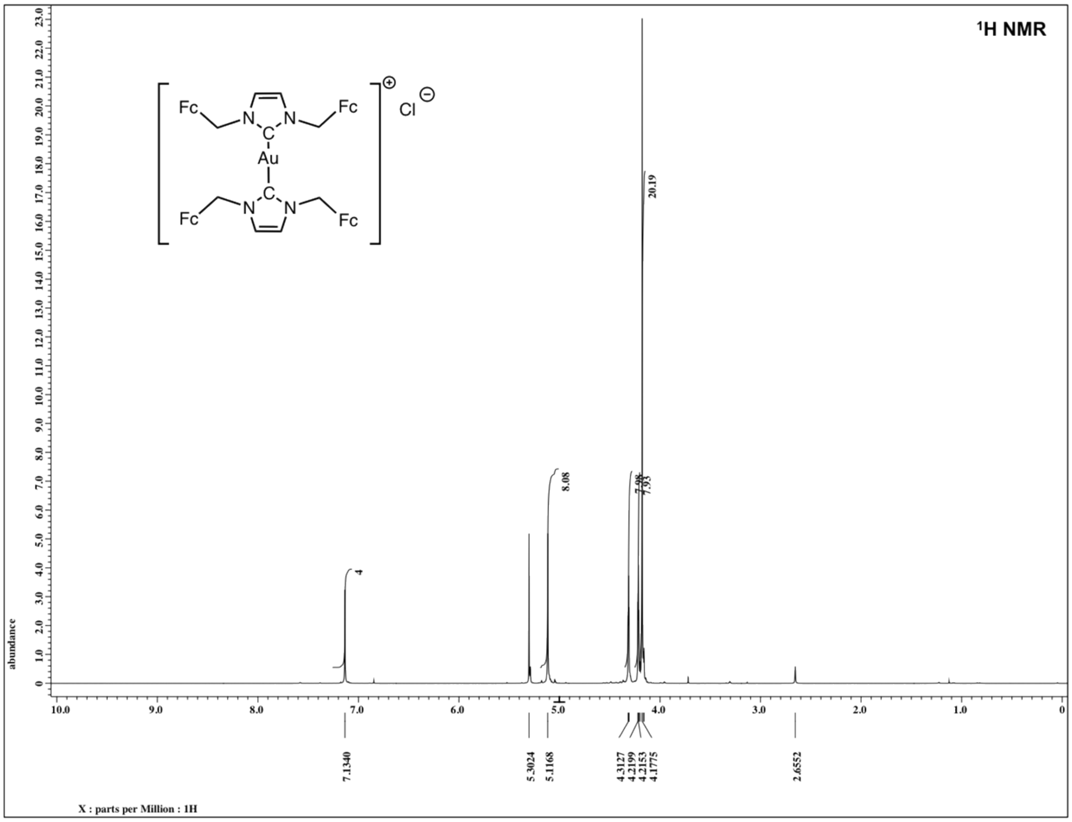

Figure S10. ^{1}H NMR spectrum of compound **6**.

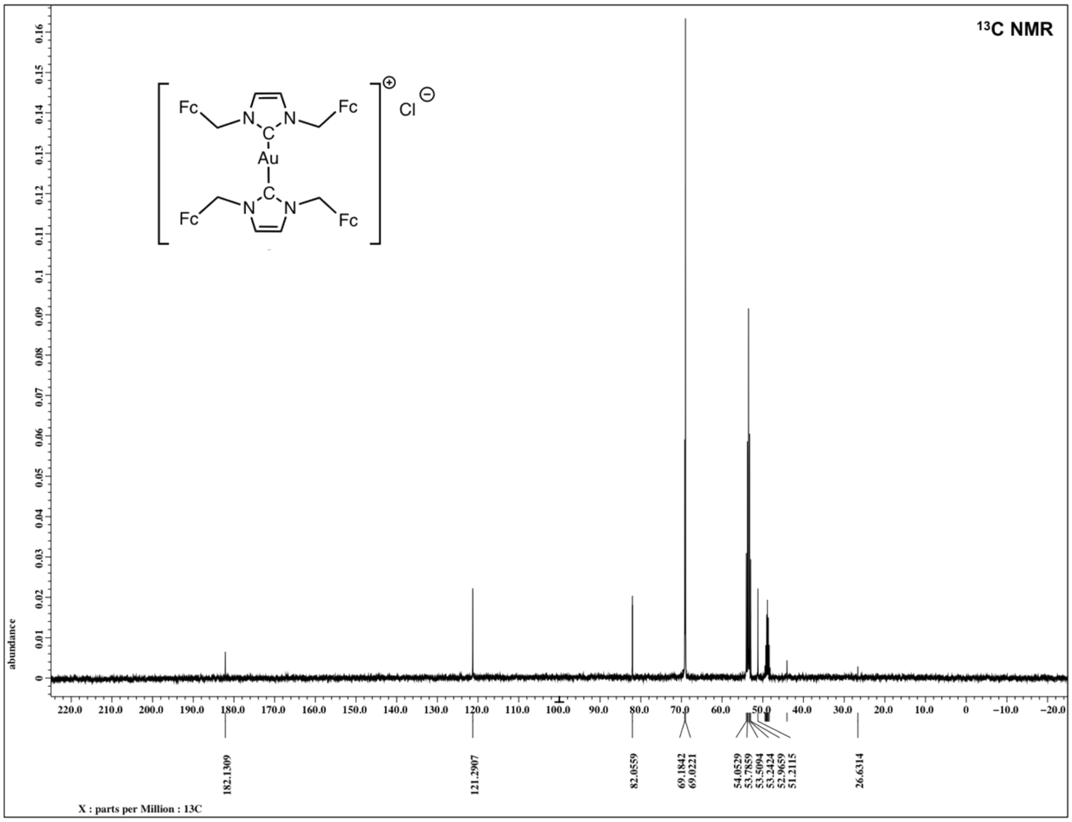

Figure S11. ^{13}C NMR spectrum of compound **6**.

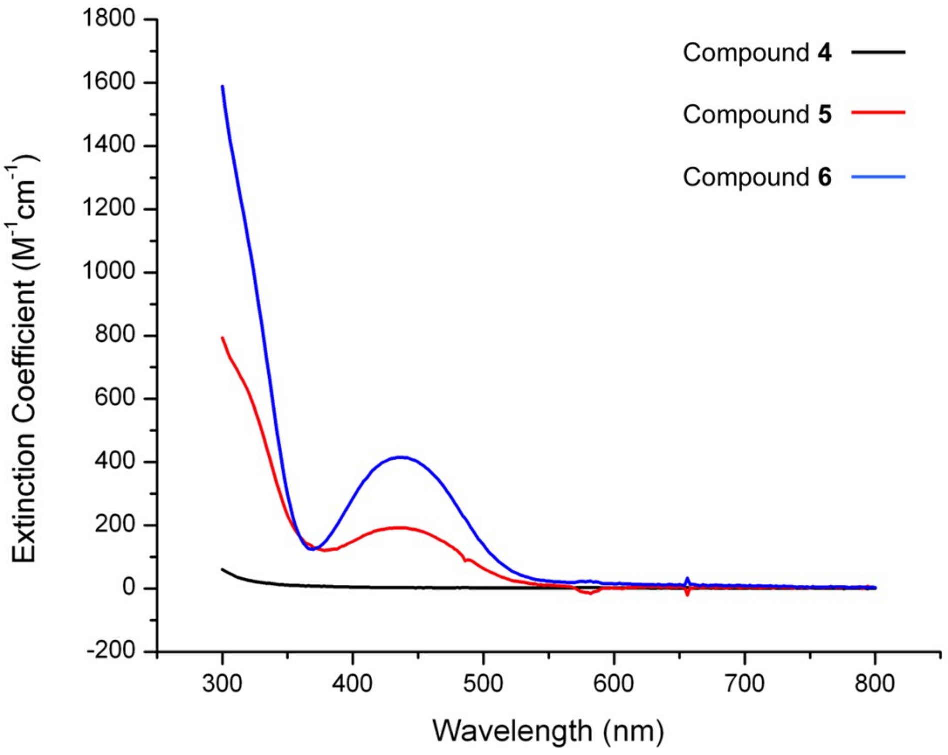

Figure S12. Electronic absorption spectra of compounds **4**, **5**, and **6** recorded in CH_2Cl_2 at 25 °C.

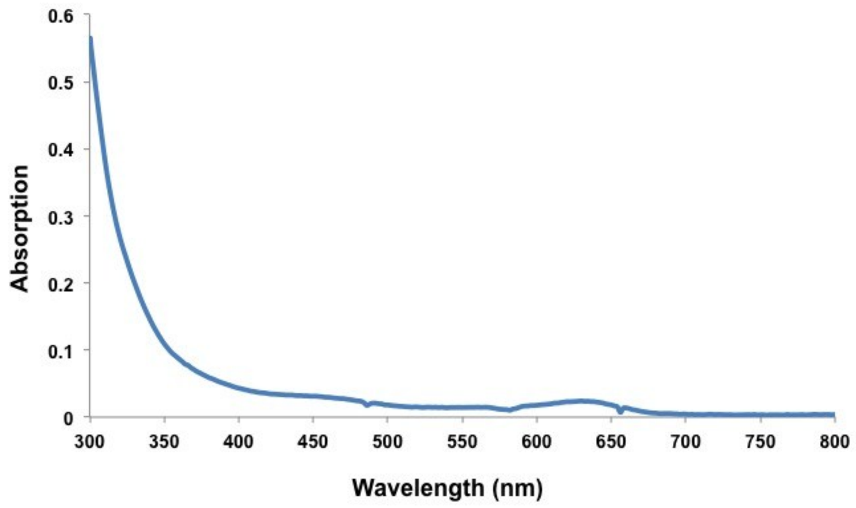

Figure S13. Electronic absorption spectra of compound **5[BF$_4$]$_2$**, recorded in CH_2Cl_2 at 25 °C.

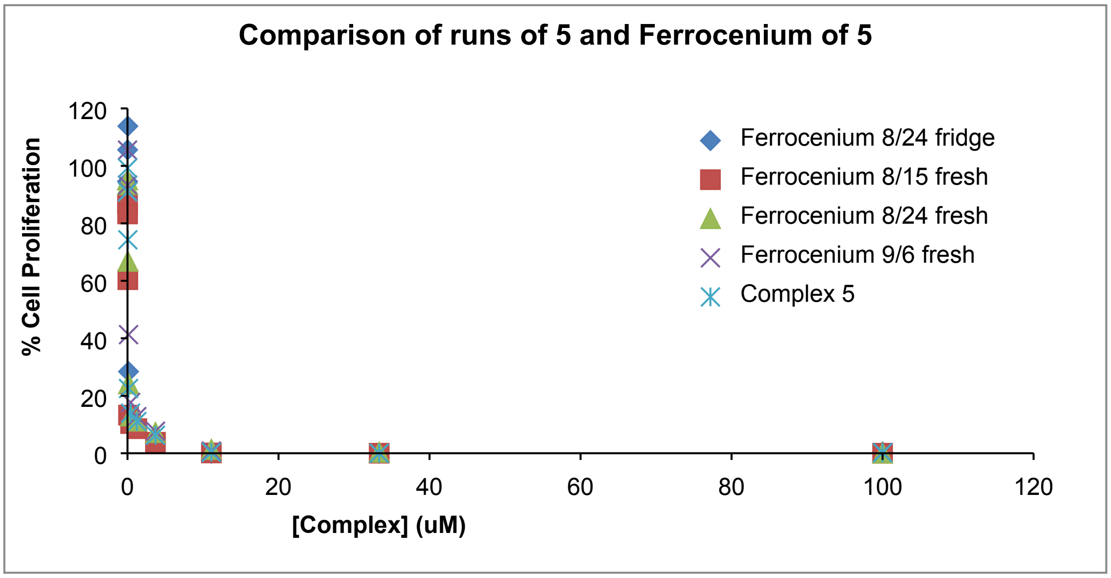

Figure S14. Cell proliferation study of ferrocene complex **5** and ferrocenium complex ([**5**][BF4$_4$]$_2$) with A549 lung cancer cells. As discussed in the main text, this study was taken as evidence that there is little to no observed cytotoxicity difference between complex **5** and its oxidized form.

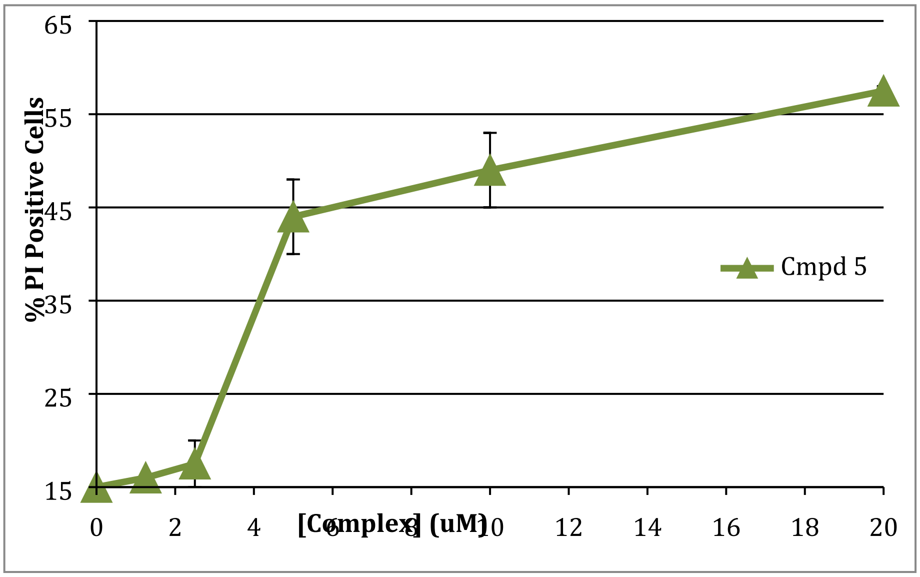

Figure S15. Positive propidium iodide (PI) signal from live A549 cells exposed to complex **5** for 4 hours. This graph illustrates the threshold of complex exposure allowed to study cellular function without stressing/killing cells.

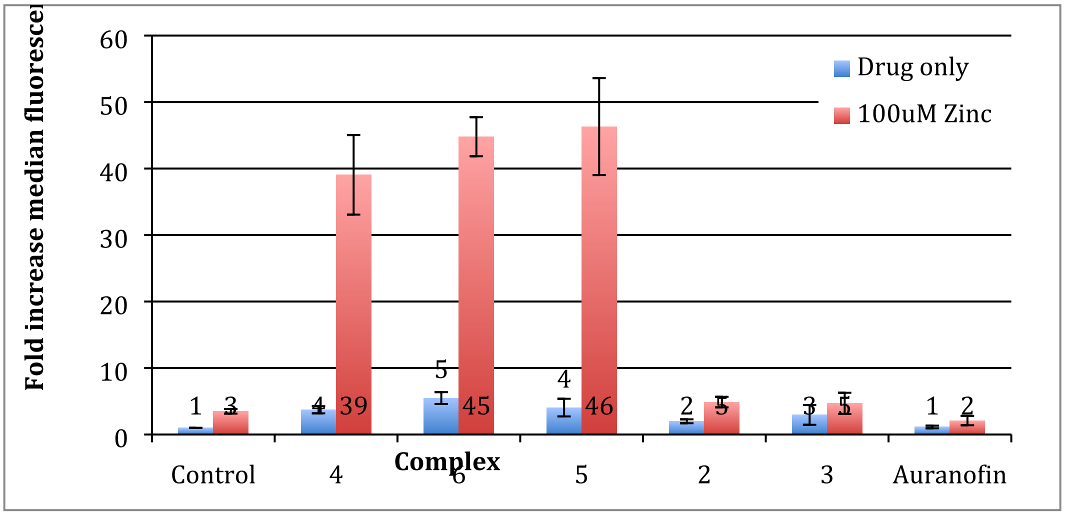

Figure S16. Difference (plotted as the fold increase) in fluorescence emission intensity as detected by flow cytometry of live A549 cells treated with 2.5 μM of complex **6** (blue) and 2.5 μM of complex **6** in the presence of 100 μM zinc acetate.

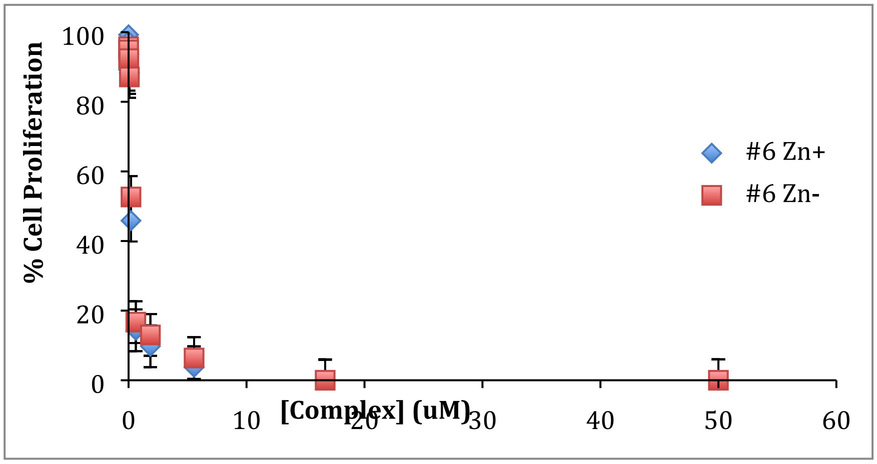

Compound(s)	IC50 value	standard deviation of IC50 value
**6**	0.1593083	0.04271
**6** + Zn	0.127415	0.051826

Figure S17. Cell proliferation study in A549 lung cancer cells comparing the activity of complex **6** vs. complex **6** in the presence of 100 μM zinc acetate.

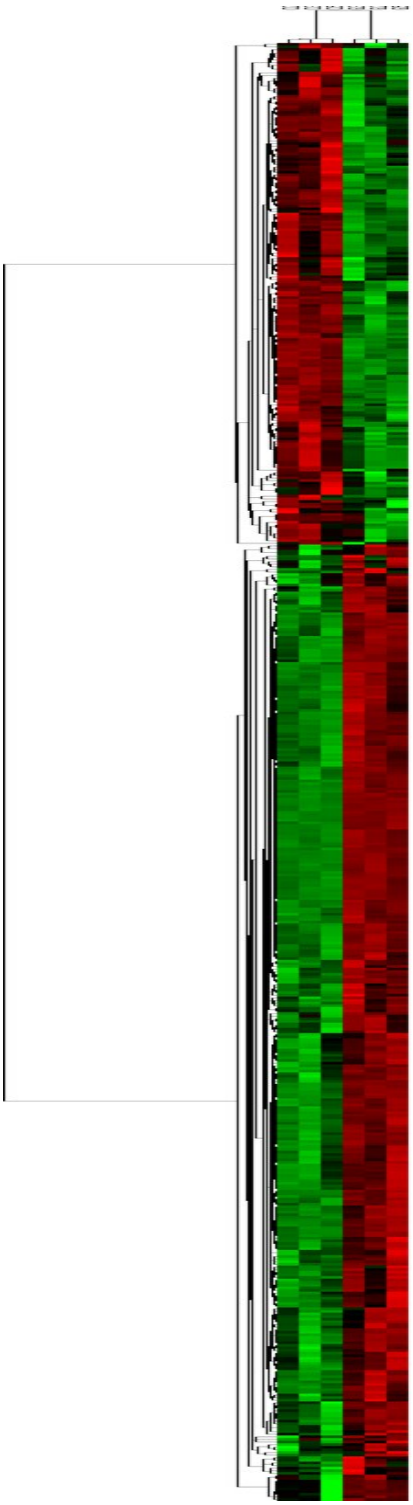

Figure S18. RNA microarray heat map illustrating differential gene expression of RNA transcripts in A549 lung cancer cells treated with vehicle (left panel) and complex **6** (right panel).

Table S2. Differential gene expression in A549 lung cancer cells treated with complex **6.**

Gene ID	Gene symbol	Gene Description	FC	P-value
79094	CHAC1	ChaC, cation transport regulator homolog 1	5.56	1.19205E-07
1649	DDIT3	DNA-damage-inducible transcript 3	4.43	4.30297E-09
57761	TRIB3	tribbles pseudokinase 3 (TRIB3)	4.41	6.5417E-08
440	ASNS	asparagine synthetase (glutamine-hydrolyzing)	3.94	3.28201E-08
27063	ANKRD1	ankyrin repeat domain 1 (cardiac muscle)	3.90	0.000102275
8614	STC2	stanniocalcin 2	3.54	1.49565E-06
116496	FAM129A	family with sequence similarity 129, member A	3.19	0.000205081
83667	SESN2	sestrin 2	3.10	1.87328E-07
29968	PSAT1	phosphoserine aminotransferase 1	2.96	4.0621E-07
80329	ULBP1	UL16 binding protein 1	2.96	1.36486E-07
9518	GDF15	growth differentiation factor 15	2.90	9.19533E-06
286343	LURAP1L	Homo sapiens leucine rich adaptor protein 1-like (LURAP1L), mRNA.	2.88	5.78279E-08
7779	SLC30A1	solute carrier family 30 (zinc transporter)	2.86	0.002992119
23645	PPP1R15A	protein phosphatase 1, regulatory subunit 15A	2.79	7.03697E-07
80201	HKDC1	Homo sapiens hexokinase domain containing 1 (HKDC1), mRNA.	2.68	8.21795E-07
10797	MTHFD2	Homo sapiens methylenetetrahydrofolate dehydrogenase (NADP+ dependent) 2, methenyltetrahydrofolate cyclohydrolase (MTHFD2), transcript variant 1, mRNA.	2.65	9.252E-08
7436	VLDLR	very low density lipoprotein receptor	2.56	3.7443E-06
26227	PHGDH	Homo sapiens phosphoglycerate dehydrogenase (PHGDH), mRNA.	2.53	1.76306E-06
4783	NFIL3	Homo sapiens nuclear factor, interleukin 3 regulated (NFIL3), transcript variant 3, mRNA.	2.52	2.14704E-05
9709	HERPUD1	homocysteine-inducible, endoplasmic reticulum stress-inducible, ubiquitin-like domain	2.48	2.53382E-07
2081	ERN1	endoplasmic reticulum to nucleus signaling 1	2.44	1.02427E-06
3162	HMOX1	heme oxygenase (decycling) 1	2.41	6.63288E-05
6509	SLC1A4	Homo sapiens solute carrier family 1 (glutamate/neutral amino acid transporter), member 4 (SLC1A4), transcript variant 2, mRNA.	2.30	1.48838E-06
2617	GARS	Homo sapiens glycyl-tRNA synthetase (GARS), mRNA.	2.27	1.23327E-06
162394	SLFN5	Homo sapiens schlafen family member 5 (SLFN5), mRNA.	2.26	2.68813E-05
80763	SPX	Homo sapiens spexin hormone (SPX), mRNA.	2.26	0.002437248
4495	MT1G	metallothionein 1G	2.24	0.034444693
833	CARS	Homo sapiens cysteinyl-tRNA synthetase (CARS), transcript variant 3, mRNA.	2.22	2.65564E-07
84962	AJUBA	Homo sapiens ajuba LIM protein (AJUBA), transcript variant 1, mRNA.	2.15	1.47549E-05

6541	SLC7A1	Homo sapiens solute carrier family 7 (cationic amino acid transporter, y+ system), member 1 (SLC7A1), mRNA.	2.15	6.79224E-06
54206	ERRFI1	Homo sapiens ERBB receptor feedback inhibitor 1 (ERRFI1), mRNA.	2.10	2.43721E-05
467	ATF3	activating transcription factor 3	2.10	1.231E-06
163732	CITED4	Homo sapiens Cbp/p300-interacting transactivator, with Glu/Asp-rich carboxy-terminal domain, 4 (CITED4), mRNA.	2.07	2.95107E-05
80008	TMEM156	Homo sapiens transmembrane protein 156 (TMEM156), mRNA.	2.06	0.001612019
1847	DUSP5	Homo sapiens dual specificity phosphatase 5 (DUSP5), mRNA.	2.04	8.75262E-06
54407	SLC38A2	Homo sapiens solute carrier family 38, member 2 (SLC38A2), mRNA.	2.04	0.001170623
2805	GOT1	Homo sapiens glutamic-oxaloacetic transaminase 1, soluble (GOT1), mRNA.	2.04	7.7059E-06
160428	ALDH1L2	Homo sapiens aldehyde dehydrogenase 1 family, member L2 (ALDH1L2), transcript variant 1, mRNA.	2.04	4.25667E-06
16	AARS	alanyl-tRNA synthetase	2.03	3.39778E-06
26136	TES	Homo sapiens testis derived transcript (3 LIM domains) (TES), transcript variant 1, mRNA.	2.02	1.71774E-06
116442	RAB39B	Homo sapiens RAB39B, member RAS oncogene family (RAB39B), mRNA.	2.02	0.002026708
9242	MSC	Homo sapiens musculin (MSC), mRNA.	2.02	3.42236E-05
255394	TCP11L2	Homo sapiens t-complex 11, testis-specific-like 2 (TCP11L2), transcript variant 1, mRNA.	2.02	1.95713E-05
586	BCAT1	Homo sapiens branched chain amino-acid transaminase 1, cytosolic (BCAT1), transcript variant 2, mRNA.	2.01	7.50148E-05
1647	GADD45A	Homo sapiens growth arrest and DNA-damage-inducible, alpha (GADD45A), transcript variant 2, mRNA.	2.00	3.37849E-05
5106	PCK2	Homo sapiens phosphoenolpyruvate carboxykinase 2 (mitochondrial) (PCK2), transcript variant 2, mRNA.	1.99	1.61087E-06
133746	JMY	Homo sapiens junction mediating and regulatory protein, p53 cofactor (JMY), mRNA.	1.98	3.0881E-05
51175	TUBE1	Homo sapiens tubulin, epsilon 1 (TUBE1), mRNA.	1.98	1.08676E-05
54541	DDIT4	Homo sapiens DNA-damage-inducible transcript 4 (DDIT4), mRNA.	1.97	2.99663E-06
1106	CHD2	Homo sapiens chromodomain helicase DNA binding protein 2 (CHD2), transcript variant 2, mRNA.	1.95	8.22031E-05
2113	ETS1	Homo sapiens v-ets avian erythroblastosis virus E26 oncogene homolog 1 (ETS1), transcript variant 1, mRNA.	1.93	0.0002419
6301	SARS	Homo sapiens seryl-tRNA synthetase (SARS),	1.93	5.4855E-06

		transcript variant 1, mRNA.		
2920	CXCL2	chemokine (C-X-C motif) ligand 2	1.93	0.00014337
4490	MT1B	metallothionein 1B	1.92	0.032952383
3576	CXCL8	chemokine (C-X-C motif) ligand 8	1.92	1.10178E-05
145788	C15orf65	Homo sapiens chromosome 15 open reading frame 65 (C15orf65), mRNA.	1.87	0.000210808
7494	XBP1	X-box binding protein 1	1.87	3.60972E-05
4141	MARS	Homo sapiens methionyl-tRNA synthetase (MARS), mRNA.	1.87	6.87998E-06
84706	GPT2	Homo sapiens glutamic pyruvate transaminase (alanine aminotransferase) 2 (GPT2), transcript variant 2, mRNA.	1.87	5.67834E-07
13	AADAC	Homo sapiens arylacetamide deacetylase (AADAC), mRNA.	1.86	1.27816E-05
1054	CEBPG	Homo sapiens CCAAT/enhancer binding protein (C/EBP), gamma (CEBPG), transcript variant 2, mRNA.	1.85	6.39341E-06
8408	ULK1	Homo sapiens unc-51 like autophagy activating kinase 1 (ULK1), mRNA.	1.84	9.42012E-06
330	BIRC3	Homo sapiens baculoviral IAP repeat containing 3 (BIRC3), transcript variant 1, mRNA.	1.83	0.000914253
3656	IRAK2	Homo sapiens interleukin-1 receptor-associated kinase 2 (IRAK2), mRNA.	1.83	5.07967E-05
23135	KDM6B	Homo sapiens lysine (K)-specific demethylase 6B (KDM6B), mRNA.	1.81	3.74662E-06
6510	SLC1A5	Homo sapiens solute carrier family 1 (neutral amino acid transporter), member 5 (SLC1A5), transcript variant 2, mRNA.	1.79	8.18401E-06
25932	CLIC4	Homo sapiens chloride intracellular channel 4 (CLIC4), mRNA.	1.78	1.9386E-05
81539	SLC38A1	Homo sapiens solute carrier family 38, member 1 (SLC38A1), transcript variant 2, mRNA.	1.77	8.50814E-05
4496	MT1H	metallothionein 1H	1.77	0.025658976
5366	PMAIP1	Homo sapiens phorbol-12-myristate-13-acetate-induced protein 1 (PMAIP1), mRNA.	1.77	0.000860403
9076	CLDN1	Homo sapiens claudin 1 (CLDN1), mRNA.	1.76	6.81326E-05
8565	YARS	Homo sapiens tyrosyl-tRNA synthetase (YARS), mRNA.	1.76	6.23673E-06
3352	HTR1D	Homo sapiens 5-hydroxytryptamine (serotonin) receptor 1D, G protein-coupled (HTR1D), mRNA.	1.75	0.000321074
54887	UHRF1BP1	Homo sapiens UHRF1 binding protein 1 (UHRF1BP1), mRNA.	1.75	1.26286E-06
54676	GTPBP2	Homo sapiens GTP binding protein 2 (GTPBP2), transcript variant 1, mRNA.	1.75	0.000103808
54557	SGTB	Homo sapiens small glutamine-rich tetratricopeptide	1.74	6.20701E-05

		repeat (TPR)-containing, beta (SGTB), mRNA.		
23710	GABARAPL1	Homo sapiens GABA(A) receptor-associated protein like 1 (GABARAPL1), mRNA.	1.74	6.12757E-06
51187	RSL24D1	Homo sapiens ribosomal L24 domain containing 1 (RSL24D1), mRNA.	1.73	6.36195E-05
1503	CTPS1	Homo sapiens CTP synthase 1 (CTPS1), mRNA.	1.72	2.01453E-05
9258	MFHAS1	Homo sapiens malignant fibrous histiocytoma amplified sequence 1 (MFHAS1), mRNA.	1.71	2.23067E-05
390	RND3	Homo sapiens Rho family GTPase 3 (RND3), transcript variant 1, mRNA.	1.70	5.84484E-06
11260	XPOT	Homo sapiens exportin, tRNA (XPOT), mRNA.	1.70	3.98803E-05
23327	NEDD4L	Homo sapiens neural precursor cell expressed, developmentally down-regulated 4-like, E3 ubiquitin protein ligase (NEDD4L), transcript variant b, mRNA.	1.68	0.000190624
56892	C8orf4	Homo sapiens chromosome 8 open reading frame 4 (C8orf4), mRNA.	1.68	0.001644041
8501	SLC43A1	Homo sapiens solute carrier family 43 (amino acid system L transporter), member 1 (SLC43A1), transcript variant 2, mRNA.	1.67	0.000939908
2673	GFPT1	Homo sapiens glutamine--fructose-6-phosphate transaminase 1 (GFPT1), transcript variant 1, mRNA.	1.66	0.000565131
3475	IFRD1	Homo sapiens interferon-related developmental regulator 1 (IFRD1), transcript variant 2, mRNA.	1.66	3.09525E-06
1848	DUSP6	Homo sapiens dual specificity phosphatase 6 (DUSP6), transcript variant 1, mRNA.	1.66	4.87461E-06
201651	AADACP1	Homo sapiens arylacetamide deacetylase pseudogene 1 (AADACP1), non-coding RNA.	1.65	0.000543321
374	AREG	Homo sapiens amphiregulin (AREG), mRNA.	1.65	9.32866E-05
2119	ETV5	Homo sapiens ets variant 5 (ETV5), mRNA.	1.65	3.551E-06
9682	KDM4A	Homo sapiens lysine (K)-specific demethylase 4A (KDM4A), mRNA.	1.65	4.3198E-05
91694	LONRF1	Homo sapiens LON peptidase N-terminal domain and ring finger 1 (LONRF1), mRNA.	1.64	1.75383E-05
143872	ARHGAP42	Homo sapiens Rho GTPase activating protein 42 (ARHGAP42), mRNA.	1.64	0.001036134
55034	MOCOS	Homo sapiens molybdenum cofactor sulfurase (MOCOS), mRNA.	1.63	0.000539505
3311	HSPA7	heat shock 70kDa protein 7	1.63	0.008645638
1956	EGFR	Homo sapiens epidermal growth factor receptor (EGFR), transcript variant 1, mRNA.	1.63	0.000388801
54532	USP53	Homo sapiens ubiquitin specific peptidase 53 (USP53), mRNA.	1.62	0.004710235
1843	DUSP1	Homo sapiens dual specificity phosphatase 1 (DUSP1), mRNA.	1.61	5.83679E-05
56907	SPIRE1	Homo sapiens spire-type actin nucleation factor 1	1.61	6.33854E-06

		(SPIRE1), transcript variant 1, mRNA.		
100506658	OCLN	Homo sapiens occludin (OCLN), transcript variant 3, mRNA.	1.61	0.002171762
22822	PHLDA1	Homo sapiens pleckstrin homology-like domain, family A, member 1 (PHLDA1), mRNA.	1.61	0.000711201
81631	MAP1LC3B	Homo sapiens microtubule-associated protein 1 light chain 3 beta (MAP1LC3B), mRNA.	1.61	0.000163794
875	CBS	Homo sapiens cystathionine-beta-synthase (CBS), transcript variant 1, mRNA.	1.61	0.000225763
9975	NR1D2	Homo sapiens nuclear receptor subfamily 1, group D, member 2 (NR1D2), transcript variant 2, mRNA.	1.61	0.000226438
64283	ARHGEF28	Homo sapiens Rho guanine nucleotide exchange factor (GEF) 28 (ARHGEF28), transcript variant 1, mRNA.	1.60	5.39283E-05
4084	MXD1	Homo sapiens MAX dimerization protein 1 (MXD1), transcript variant 2, mRNA.	1.60	1.45459E-05
2069	EREG	Homo sapiens epiregulin (EREG), mRNA.	1.60	0.001269116
468	ATF4	Homo sapiens activating transcription factor 4 (ATF4), transcript variant 1, mRNA.	1.59	2.85502E-05
59277	NTN4	Homo sapiens netrin 4 (NTN4), mRNA.	1.59	0.000121864
5054	SERPINE1	Homo sapiens serpin peptidase inhibitor, clade E (nexin, plasminogen activator inhibitor type 1), member 1 (SERPINE1), mRNA.	1.59	0.027882965
57522	SRGAP1	Homo sapiens SLIT-ROBO Rho GTPase activating protein 1 (SRGAP1), mRNA.	1.59	0.001259494
3219	HOXB9	Homo sapiens homeobox B9 (HOXB9), mRNA.	1.59	0.000868323
4335	MNT	Homo sapiens MAX network transcriptional repressor (MNT), mRNA.	1.58	0.000126869
3673	ITGA2	Homo sapiens integrin, alpha 2 (CD49B, alpha 2 subunit of VLA-2 receptor) (ITGA2), transcript variant 1, mRNA.	1.58	0.001464622
51315	KRCC1	Homo sapiens lysine-rich coiled-coil 1 (KRCC1), mRNA.	1.58	0.002096546
9181	ARHGEF2	Homo sapiens Rho/Rac guanine nucleotide exchange factor (GEF) 2 (ARHGEF2), transcript variant 1, mRNA.	1.58	7.81428E-05
8682	PEA15	Homo sapiens phosphoprotein enriched in astrocytes 15 (PEA15), transcript variant 2, mRNA.	1.58	2.52053E-05
100507178	SLFNL1-AS1	Homo sapiens SLFNL1 antisense RNA 1 (SLFNL1-AS1), long non-coding RNA.	1.57	0.00098649
118460	EXOSC6	Homo sapiens exosome component 6 (EXOSC6), mRNA.	1.57	0.000125254
5074	PAWR	Homo sapiens PRKC, apoptosis, WT1, regulator (PAWR), mRNA.	1.57	0.00078233
26959	HBP1	Homo sapiens HMG-box transcription factor 1 (HBP1), transcript variant 1, mRNA.	1.57	0.000504964

26471	NUPR1	Homo sapiens nuclear protein, transcriptional regulator, 1 (NUPR1), transcript variant 1, mRNA.	1.57	0.00034291
80853	KDM7A	Homo sapiens lysine (K)-specific demethylase 7A (KDM7A), mRNA.	1.56	3.48311E-05
8795	TNFRSF10B	Homo sapiens tumor necrosis factor receptor superfamily, member 10b (TNFRSF10B), transcript variant 1, mRNA.	1.56	1.19697E-05
7422	VEGFA	Homo sapiens vascular endothelial growth factor A (VEGFA), transcript variant 1, mRNA.	1.56	7.97779E-06
639	PRDM1	Homo sapiens PR domain containing 1, with ZNF domain (PRDM1), transcript variant 1, mRNA.	1.56	6.02793E-05
114880	OSBPL6	Homo sapiens oxysterol binding protein-like 6 (OSBPL6), transcript variant 3, mRNA.	1.56	0.001492447
6374	CXCL5	Homo sapiens chemokine (C-X-C motif) ligand 5 (CXCL5), mRNA.	1.56	0.000268505
54498	SMOX	Homo sapiens spermine oxidase (SMOX), transcript variant 1, mRNA.	1.55	0.001642205
100422943	MIR3189	Homo sapiens microRNA 3189 (MIR3189), microRNA.	1.55	0.001025243
10468	FST	Homo sapiens follistatin (FST), transcript variant FST317, mRNA.	1.55	1.5755E-05
2697	GJA1	Homo sapiens gap junction protein, alpha 1, 43kDa (GJA1), mRNA.	1.55	0.000935992
80315	CPEB4	Homo sapiens cytoplasmic polyadenylation element binding protein 4 (CPEB4), mRNA.	1.55	0.000446575
10150	MBNL2	Homo sapiens muscleblind-like splicing regulator 2 (MBNL2), transcript variant 1, mRNA.	1.55	0.002781866
205860	TRIML2	Homo sapiens tripartite motif family-like 2 (TRIML2), mRNA.	1.54	0.000126615
6782	HSPA13	heat shock protein 70kDa family, member 13	1.54	0.001471724
1490	CTGF	Homo sapiens connective tissue growth factor (CTGF), mRNA.	1.54	0.00160884
81788	NUAK2	Homo sapiens NUAK family, SNF1-like kinase, 2 (NUAK2), mRNA.	1.54	0.004031124
2887	GRB10	Homo sapiens growth factor receptor-bound protein 10 (GRB10), transcript variant 2, mRNA.	1.54	6.04107E-05
6536	SLC6A9	Homo sapiens solute carrier family 6 (neurotransmitter transporter, glycine), member 9 (SLC6A9), transcript variant 3, mRNA.	1.53	4.48892E-05
1407	CRY1	Homo sapiens cryptochrome circadian clock 1 (CRY1), mRNA.	1.53	8.38714E-05
9853	RUSC2	Homo sapiens RUN and SH3 domain containing 2 (RUSC2), transcript variant 1, mRNA.	1.53	0.000732852
8780	RIOK3	Homo sapiens RIO kinase 3 (RIOK3), mRNA.	1.53	0.001933866
3491	CYR61	Homo sapiens cysteine-rich, angiogenic inducer, 61 (CYR61), mRNA.	1.53	0.012661708

4616	GADD45B	Homo sapiens growth arrest and DNA-damage-inducible, beta (GADD45B), mRNA.	1.53	0.001441005
6897	TARS	Homo sapiens threonyl-tRNA synthetase (TARS), transcript variant 1, mRNA.	1.52	7.61352E-05
6515	SLC2A3	Homo sapiens solute carrier family 2 (facilitated glucose transporter), member 3 (SLC2A3), mRNA.	1.52	0.005525219
79836	LONRF3	Homo sapiens LON peptidase N-terminal domain and ring finger 3 (LONRF3), transcript variant 1, mRNA.	1.51	0.000183789
3309	HSPA5	heat shock 70kDa protein 5 (glucose-regulated protein, 78kDa)	1.51	2.83009E-05
407021	MIR29A	Homo sapiens microRNA 29a (MIR29A), microRNA.	1.51	0.000616807
100859930	HEIH	Homo sapiens hepatocellular carcinoma up-regulated EZH2-associated long non-coding RNA (HEIH), long non-coding RNA.	1.51	0.000176998
26511	CHIC2	Homo sapiens cysteine-rich hydrophobic domain 2 (CHIC2), mRNA.	1.51	0.000607613
283991	UBALD2	Homo sapiens UBA-like domain containing 2 (UBALD2), mRNA.	1.51	0.000335073
326343	MT1DP	Homo sapiens metallothionein 1D, pseudogene (MT1DP), transcript variant 1, non-coding RNA.	1.50	0.03870575
2058	EPRS	Homo sapiens glutamyl-prolyl-tRNA synthetase (EPRS), mRNA.	1.49	0.001311123
114915	EPB41L4A-AS1	Homo sapiens EPB41L4A antisense RNA 1 (EPB41L4A-AS1), long non-coding RNA.	1.49	0.00015942
1051	CEBPB	Homo sapiens CCAAT/enhancer binding protein (C/EBP), beta (CEBPB), transcript variant 1, mRNA.	1.49	0.003739844
694	BTG1	Homo sapiens B-cell translocation gene 1, anti-proliferative (BTG1), mRNA.	1.48	0.00029834
80709	AKNA	Homo sapiens AT-hook transcription factor (AKNA), mRNA.	1.48	0.000172609
4677	NARS	Homo sapiens asparaginyl-tRNA synthetase (NARS), mRNA.	1.48	0.000213934
7453	WARS	Homo sapiens tryptophanyl-tRNA synthetase (WARS), transcript variant 1, mRNA.	1.48	0.000373972
4609	MYC	Homo sapiens v-myc avian myelocytomatosis viral oncogene homolog (MYC), mRNA.	1.48	9.19855E-05
283337	ZNF740	Homo sapiens zinc finger protein 740 (ZNF740), mRNA.	1.48	1.7541E-05
29948	OSGIN1	oxidative stress induced growth inhibitor 1	1.48	0.000144965
5743	PTGS2	Homo sapiens prostaglandin-endoperoxide synthase 2 (prostaglandin G/H synthase and cyclooxygenase) (PTGS2), mRNA.	1.48	0.010842375
25902	MTHFD1L	Homo sapiens methylenetetrahydrofolate dehydrogenase (NADP+ dependent) 1-like (MTHFD1L), transcript variant 1, mRNA.	1.48	0.00021717
57820	CCNB1IP1	Homo sapiens cyclin B1 interacting protein 1, E3	1.48	5.26928E-05

		ubiquitin protein ligase (CCNB1IP1), transcript variant 1, mRNA.		
5723	PSPH	Homo sapiens phosphoserine phosphatase (PSPH), mRNA.	1.47	0.000154642
54977	SLC25A38	Homo sapiens solute carrier family 25, member 38 (SLC25A38), mRNA.	1.47	0.000552094
27250	PDCD4	Homo sapiens programmed cell death 4 (neoplastic transformation inhibitor) (PDCD4), transcript variant 3, mRNA.	1.47	0.001169422
55659	ZNF416	Homo sapiens zinc finger protein 416 (ZNF416), mRNA.	1.46	4.87738E-05
4864	NPC1	Homo sapiens Niemann-Pick disease, type C1 (NPC1), mRNA.	1.46	0.00176907
84798	C19orf48	Homo sapiens chromosome 19 open reading frame 48 (C19orf48), transcript variant 1, mRNA.	1.46	0.000619345
7975	MAFK	Homo sapiens v-maf avian musculoaponeurotic fibrosarcoma oncogene homolog K (MAFK), mRNA.	1.46	2.55415E-05
571	BACH1	Homo sapiens BTB and CNC homology 1, basic leucine zipper transcription factor 1 (BACH1), transcript variant 2, mRNA.	1.46	0.003056492
558	AXL	Homo sapiens AXL receptor tyrosine kinase (AXL), transcript variant 2, mRNA.	1.46	0.001316471
144402	CPNE8	Homo sapiens copine VIII (CPNE8), mRNA.	1.45	0.000104847
5271	SERPINB8	Homo sapiens serpin peptidase inhibitor, clade B (ovalbumin), member 8 (SERPINB8), transcript variant 3, mRNA.	1.45	9.79701E-05
1778	DYNC1H1	Homo sapiens dynein, cytoplasmic 1, heavy chain 1 (DYNC1H1), mRNA.	1.45	0.001315251
7706	TRIM25	Homo sapiens tripartite motif containing 25 (TRIM25), mRNA.	1.45	0.000473856
8535	CBX4	Homo sapiens chromobox homolog 4 (CBX4), mRNA.	1.45	0.000520384
3976	LIF	Homo sapiens leukemia inhibitory factor (LIF), transcript variant 1, mRNA.	1.45	0.00021215
55596	ZCCHC8	Homo sapiens zinc finger, CCHC domain containing 8 (ZCCHC8), mRNA.	1.45	0.002333212
10469	TIMM44	Homo sapiens translocase of inner mitochondrial membrane 44 homolog (yeast) (TIMM44), mRNA.	1.45	2.8389E-05
96459	FNIP1	Homo sapiens folliculin interacting protein 1 (FNIP1), transcript variant 2, mRNA.	1.44	0.001006678
8061	FOSL1	Homo sapiens FOS-like antigen 1 (FOSL1), mRNA.	1.44	0.001483349
9411	ARHGAP29	Homo sapiens Rho GTPase activating protein 29 (ARHGAP29), mRNA.	1.44	0.002264516
63874	ABHD4	Homo sapiens abhydrolase domain containing 4 (ABHD4), mRNA.	1.44	0.00011778
9590	AKAP12	Homo sapiens A kinase (PRKA) anchor protein 12 (AKAP12), transcript variant 1, mRNA.	1.44	0.005606399

1604	CD55	Homo sapiens CD55 molecule, decay accelerating factor for complement (Cromer blood group) (CD55), transcript variant 1, mRNA.	1.44	0.002217375
4498	MT1JP	Homo sapiens metallothionein 1J, pseudogene (MT1JP), non-coding RNA.	1.44	0.020411769
3376	IARS	Homo sapiens isoleucyl-tRNA synthetase (IARS), transcript variant 1, mRNA.	1.44	4.26376E-05
6472	SHMT2	Homo sapiens serine hydroxymethyltransferase 2 (mitochondrial) (SHMT2), transcript variant 2, mRNA.	1.44	0.000183891
6868	ADAM17	Homo sapiens ADAM metallopeptidase domain 17 (ADAM17), mRNA.	1.44	0.000558432
101929467	LURAP1L-AS1	havana:known chromosome:GRCh38:9:12700100:12814345:-1 gene:ENSG00000235448 gene_biotype:antisense transcript_biotype:antisense	1.44	0.001039004
148189	LINC00662	Homo sapiens long intergenic non-protein coding RNA 662 (LINC00662), long non-coding RNA.	1.43	0.002764419
8894	EIF2S2	Homo sapiens eukaryotic translation initiation factor 2, subunit 2 beta, 38kDa (EIF2S2), mRNA.	1.43	0.000618528
58526	MID1IP1	Homo sapiens MID1 interacting protein 1 (MID1IP1), transcript variant 2, mRNA.	1.43	6.92004E-05
6617	SNAPC1	Homo sapiens small nuclear RNA activating complex, polypeptide 1, 43kDa (SNAPC1), mRNA.	1.43	0.005124127
10193	RNF41	Homo sapiens ring finger protein 41, E3 ubiquitin protein ligase (RNF41), transcript variant 4, mRNA.	1.43	0.000232822
23345	SYNE1	Homo sapiens spectrin repeat containing, nuclear envelope 1 (SYNE1), transcript variant 2, mRNA.	1.43	0.000403164
4253	CTAGE5	Homo sapiens CTAGE family, member 5 (CTAGE5), transcript variant 5, mRNA.	1.43	0.000636654
79745	CLIP4	Homo sapiens CAP-GLY domain containing linker protein family, member 4 (CLIP4), transcript variant 1, mRNA.	1.43	5.91731E-05
60370	AVPI1	Homo sapiens arginine vasopressin-induced 1 (AVPI1), mRNA.	1.42	0.000221634
27	ABL2	Homo sapiens ABL proto-oncogene 2, non-receptor tyrosine kinase (ABL2), transcript variant d, mRNA.	1.42	0.00440369
190	NR0B1	Homo sapiens nuclear receptor subfamily 0, group B, member 1 (NR0B1), mRNA.	1.42	0.00092319
169792	GLIS3	Homo sapiens GLIS family zinc finger 3 (GLIS3), transcript variant 1, mRNA.	1.42	6.3173E-05
7693	ZNF134	Homo sapiens zinc finger protein 134 (ZNF134), mRNA.	1.42	0.000554218
122553	TRAPPC6B	Homo sapiens trafficking protein particle complex 6B (TRAPPC6B), transcript variant 1, mRNA.	1.42	0.001304266
121268	RHEBL1	Homo sapiens Ras homolog enriched in brain like 1 (RHEBL1), mRNA.	1.42	0.000241942

127544	RNF19B	Homo sapiens ring finger protein 19B (RNF19B), transcript variant 2, mRNA.	1.41	0.00082868
10123	ARL4C	Homo sapiens ADP-ribosylation factor-like 4C (ARL4C), transcript variant 2, mRNA.	0.71	6.77608E-05
6928	HNF1B	Homo sapiens HNF1 homeobox B (HNF1B), transcript variant 1, mRNA.	0.70	0.000841571
5209	PFKFB3	Homo sapiens 6-phosphofructo-2-kinase/fructose-2,6-biphosphatase 3 (PFKFB3), transcript variant 2, mRNA.	0.70	0.000159423
64759	TNS3	Homo sapiens tensin 3 (TNS3), mRNA.	0.70	0.000143623
10137	RBM12	Homo sapiens RNA binding motif protein 12 (RBM12), transcript variant 3, mRNA.	0.70	0.000166961
10110	SGK2	Homo sapiens serum/glucocorticoid regulated kinase 2 (SGK2), transcript variant 3, mRNA.	0.70	4.90732E-05
100507012	BMPR1B-AS1	havana:known chromosome:GRCh38:4:94743800:94757533:-1 gene:ENSG00000249599 gene_biotype:lincRNA transcript_biotype:lincRNA	0.70	0.020454528
117246	FTSJ3	Homo sapiens FtsJ homolog 3 (E. coli) (FTSJ3), mRNA.	0.70	3.42647E-05
55388	MCM10	Homo sapiens minichromosome maintenance complex component 10 (MCM10), transcript variant 2, mRNA.	0.69	0.000307972
7903	ST8SIA4	Homo sapiens ST8 alpha-N-acetyl-neuraminide alpha-2,8-sialyltransferase 4 (ST8SIA4), transcript variant 1, mRNA.	0.69	0.000394193
4998	ORC1	Homo sapiens origin recognition complex, subunit 1 (ORC1), transcript variant 2, mRNA.	0.69	0.000295964
9203	ZMYM3	Homo sapiens zinc finger, MYM-type 3 (ZMYM3), transcript variant 3, mRNA.	0.69	0.001332034
3215	HOXB5	Homo sapiens homeobox B5 (HOXB5), mRNA.	0.69	0.000438995
1031	CDKN2C	Homo sapiens cyclin-dependent kinase inhibitor 2C (p18, inhibits CDK4) (CDKN2C), transcript variant 1, mRNA.	0.69	0.000542043
54443	ANLN	Homo sapiens anillin, actin binding protein (ANLN), transcript variant 1, mRNA.	0.69	0.042628236
84951	TNS4	Homo sapiens tensin 4 (TNS4), mRNA.	0.69	0.007525507
729438	GATSL2	Homo sapiens GATS protein-like 2 (GATSL2), mRNA.	0.69	4.79033E-05
388	RHOB	Homo sapiens ras homolog family member B (RHOB), mRNA.	0.68	0.013151504
23336	SYNM	Homo sapiens synemin, intermediate filament protein (SYNM), transcript variant B, mRNA.	0.68	0.000477282
91442	C19orf40	Homo sapiens chromosome 19 open reading frame 40 (C19orf40), mRNA.	0.68	0.000806062
1479	CSTF3	Homo sapiens cleavage stimulation factor, 3 pre-RNA, subunit 3, 77kDa (CSTF3), transcript variant 2, mRNA.	0.68	0.00247835
6657	SOX2	Homo sapiens SRY (sex determining region Y)-box 2	0.68	0.000708162

		(SOX2), mRNA.		
6821	SUOX	Homo sapiens sulfite oxidase (SUOX), transcript variant 1, mRNA.	0.68	7.52756E-05
23670	TMEM2	Homo sapiens transmembrane protein 2 (TMEM2), transcript variant 2, mRNA.	0.67	0.000117566
26298	EHF	Homo sapiens ets homologous factor (EHF), transcript variant 3, mRNA.	0.67	0.001355271
8335	HIST1H2AB	Homo sapiens histone cluster 1, H2ab (HIST1H2AB), mRNA.	0.66	0.000909328
57561	ARRDC3	Homo sapiens arrestin domain containing 3 (ARRDC3), mRNA.	0.66	0.000235862
899	CCNF	Homo sapiens cyclin F (CCNF), mRNA.	0.66	0.000909293
9075	CLDN2	Homo sapiens claudin 2 (CLDN2), transcript variant 2, mRNA.	0.66	0.001285849
374393	FAM111B	Homo sapiens family with sequence similarity 111, member B (FAM111B), transcript variant 2, mRNA.	0.66	0.001260666
8346	HIST1H2BI	Homo sapiens histone cluster 1, H2bi (HIST1H2BI), mRNA.	0.66	0.000358851
257629	ANKS4B	Homo sapiens ankyrin repeat and sterile alpha motif domain containing 4B (ANKS4B), mRNA.	0.65	0.011959771
406891	MIRLET7I	Homo sapiens microRNA let-7i (MIRLET7I), microRNA.	0.65	0.000402562
100126798	SNAR-A1	Homo sapiens small ILF3/NF90-associated RNA A1 (SNAR-A1), small nuclear RNA.	0.64	0.001167124
9982	FGFBP1	Homo sapiens fibroblast growth factor binding protein 1 (FGFBP1), mRNA.	0.64	0.001946405
1903	S1PR3	Homo sapiens sphingosine-1-phosphate receptor 3 (S1PR3), mRNA.	0.63	0.012657008
57181	SLC39A10	solute carrier family 39 (zinc transporter), member 10	0.63	0.016893383
10112	KIF20A	Homo sapiens kinesin family member 20A (KIF20A), mRNA.	0.61	0.003577463
283460	HNF1A-AS1	Homo sapiens HNF1A antisense RNA 1 (HNF1A-AS1), long non-coding RNA.	0.61	0.000203855
10614	HEXIM1	Homo sapiens hexamethylene bis-acetamide inducible 1 (HEXIM1), mRNA.	0.59	1.31185E-05
196410	METTL7B	Homo sapiens methyltransferase like 7B (METTL7B), mRNA.	0.58	0.000481523
23007	PLCH1	Homo sapiens phospholipase C, eta 1 (PLCH1), transcript variant 1, mRNA.	0.58	0.000402872
56829	ZC3HAV1	Homo sapiens zinc finger CCCH-type, antiviral 1 (ZC3HAV1), transcript variant 1, mRNA.	0.57	5.75866E-06
3306	HSPA2	heat shock 70kDa protein 2	0.56	1.49155E-05
57622	LRFN1	Homo sapiens leucine rich repeat and fibronectin type III domain containing 1 (LRFN1), mRNA.	0.56	0.001525524
6662	SOX9	Homo sapiens SRY (sex determining region Y)-box 9 (SOX9), mRNA.	0.52	2.30174E-05

6502	SKP2	Homo sapiens S-phase kinase-associated protein 2, E3 ubiquitin protein ligase (SKP2), transcript variant 3, mRNA.	0.51	2.1928E-06
54894	RNF43	Homo sapiens ring finger protein 43 (RNF43), mRNA.	0.48	0.000165271
100422934	MIR3143	Homo sapiens microRNA 3143 (MIR3143), microRNA.	0.45	3.61889E-06
6347	CCL2	chemokine (C-C motif)	0.39	1.53404E-05

References:

1) J. R. Aranzaes, M.-C. Daniel and D. Astruc, *Can. J. Chem.*, 2006, **84**, 288-299; I. Noviandri, K. N. Brown, D. S. Fleming, P. T. Gulyas, P. A. Lay, A. F. Masters and L. Phillips, *The J. Phys. Chem. B*, 1999, **103**, 6713-6722.
